# To Tweak or Not to Tweak. How Exploiting Flexibilities in Gene Set Analysis Leads to Overoptimism

**DOI:** 10.1002/bimj.70016

**Published:** 2024-12-19

**Authors:** Milena Wünsch, Christina Sauer, Moritz Herrmann, Ludwig Christian Hinske, Anne‐Laure Boulesteix

**Affiliations:** ^1^ Institute for Medical Information Processing, Biometry, and Epidemiology, Faculty of Medicine LMU Munich Munich Germany; ^2^ Munich Center for Machine Learning Munich Germany; ^3^ Institute for Digital Medicine University Hospital of Augsburg Augsburg Germany

**Keywords:** cherry‐picking, gene set analysis, multiplicity, overoptimism, uncertainty

## Abstract

Gene set analysis, a popular approach for analyzing high‐throughput gene expression data, aims to identify sets of genes that show enriched expression patterns between two conditions. In addition to the multitude of methods available for this task, users are typically left with many options when creating the required input and specifying the internal parameters of the chosen method. This flexibility can lead to uncertainty about the “right” choice, further reinforced by a lack of evidence‐based guidance. Especially when their statistical experience is scarce, this uncertainty might entice users to produce preferable results using a “trial‐and‐error” approach. While it may seem unproblematic at first glance, this practice can be viewed as a form of “cherry‐picking” and cause an optimistic bias, rendering the results nonreplicable on independent data. After this problem has attracted a lot of attention in the context of classical hypothesis testing, we now aim to raise awareness of such overoptimism in the different and more complex context of gene set analyses. We mimic a hypothetical researcher who systematically selects the analysis variants yielding their preferred results, thereby considering three distinct goals they might pursue. Using a selection of popular gene set analysis methods, we tweak the results in this way for two frequently used benchmark gene expression data sets. Our study indicates that the potential for overoptimism is particularly high for a group of methods frequently used despite being commonly criticized. We conclude by providing practical recommendations to counter overoptimism in research findings in gene set analysis and beyond.

## 1 Introduction

When performing gene set analysis (GSA), a researcher must decide on a suitable analysis strategy, including all analytical choices concerning the method, its internal parameter setting, and the preprocessing approach used to format the gene expression data as required by the selected method. The particular difficulty in this decision lies in the great multiplicity the researcher faces in all three aspects. Generally, the multiplicity of possible data analysis (and data collection) strategies is also referred to as *researchers' degrees of freedom* (Simmons, Nelson, and Simonsohn 2011). A thorough investigation of these researchers' degrees of freedom in GSA with a focus on analyzing the data is provided in our previous work (Wünsch et al. 2023), in which we observe that there is little guidance on most of them. This leads to a considerable *uncertainty* about the “right” or most suitable analysis strategy from the multitude of available options.

In genomics and related fields, new research findings often heavily rely on gene set analyses (Ballouz, Pavlidis, and Gillis 2017). Researchers might thus be tempted to exploit this uncertainty, that is, to choose the analysis strategy that yields the “best” or most promising results after trying out several different analysis strategies concerning the choice of the GSA method, data preprocessing approach, and internal parameters. Especially researchers with little statistical experience are often unaware that such tweaking, which appears unproblematic at first view, is a form of the questionable research practice known as *cherry‐picking*. If they *selectively report* the chosen analysis strategy and corresponding results while withholding the remaining “worse” results, the research findings are likely to be optimistically biased. As such, they may be poorly replicable based on new and independent data.

Note that the definition of the term “replicable” differs considerably across scientific fields and even between scientists and publications within the fields. See Nosek and Errington (2020) for a broad epistemological discussion of the concept. In the context of this study, we define replication as the attempt to recreate a previously obtained research finding by applying the same methods as in the original study on independent data. A research finding (or a whole study) is considered as “replicable” if the replication attempt is successful—where success can be defined in various ways depending on the substantive context and the considered data analysis methods. See, for example, the criteria proposed by Held, Micheloud, and Pawel (2022) in the context of classical statistical testing.

Failure to replicate may have various reasons. The finding of the original study may be simply a type‐1 error (in case a test is used) or an inflated effect in the absence of methodological flaw, or may result from flaws in design, implementation or analysis (Open Science Collaboration 2012). Even if flaws are not always involved in the lack of replicability of research findings, replicability is considered a core quality that all empirical research findings should fulfill. As claimed by Popper (2005), nonreplicable single findings are of “no significance to science.”

The statistical mechanisms behind the lack of replicability of research findings are well understood in the context of classical statistical testing. Ioannidis (2005) outlines that results are often presented as “conclusive” even if they were derived from only a single (potentially flawed) study, and that flexibility in the design or the analytical mode can enable researchers to transform results from negative to positive, that is, from nonsignificant to significant. It then does not come as a surprise that such results are not confirmed in later replication studies.

However, the lack of replicability of data analysis results has alarmingly drawn little attention beyond the context of statistical testing until very recently. A contribution to this topic is given in Ullmann et al. (2023), who demonstrate the mechanisms of cherry‐picking and its quantitative impact in terms of replicability in the specific context of unsupervised (clustering and network) analysis of microbiome data. In a study investigating the variability of the results generated across 13 popular GSA methods, Maleki et al. (2019) observe that the number of gene sets detected as differentially enriched differs by up to two orders of magnitude between the methods. This suggests that GSA may potentially be similarly subject to cherry‐picking mechanisms, although in a different, perhaps less decipherable manner than classical significance testing or unsupervised analysis. An assessment of the impact of cherry‐picking in GSA taking all types of degrees of freedom into account is still pending. The present study aims to fill this gap.

More precisely, we quantitatively illustrate the questionable research practices that lead to overoptimistic (and therefore nonreplicable) results in the context of GSA using real gene expression data sets. In our study, we imitate hypothetical researchers tweaking the GSA results by exploiting the inherent uncertainty about the analytical choices. We thereby proceed in a *stepwise* manner, reflecting the typical approach of researchers who are unaware of the impact of cherry‐picking yet fundamentally well intentioned. Using real gene expression data sets, we mimic their search for the “best” results across a wide variety of analytical choices for seven popular GSA methods, considering successively three different goals they might pursue. In particular, we investigate settings in which no gene sets are expected to be detected as differentially enriched so that the achievement of any statistically significant results through the modification of the analysis strategy can be directly interpreted as overoptimism.

This paper is structured as follows. We elaborate on the connection between the inherent uncertainty in the choice of the analysis strategy in GSA and overoptimism in Section 2. In Section 3, we describe the design of our study to assess the potential of GSA to generate overoptimistic and therefore nonreplicable research findings, followed by the results in Section 4. Finally, we provide a discussion together with guidance to prevent overoptimism in Section 5.

## 2 From Uncertainty to Overoptimism in Gene Set Analysis

When selecting an appropriate analysis strategy to perform GSA, a researcher faces a noteworthy number of choices. This can lead to a considerable *uncertainty* about the “right” (i.e., most suitable) choice among the corresponding options. Note that this uncertainty is to be distinguished from the uncertainty about *which practical steps are generally necessary* when carrying out GSA. In our study, we assume that the user knows which steps are required to run GSA, but in each of these steps, they face a variety of options that lead to uncertainty about the right choice.

In the following, we are guided by the work of Hoffmann et al. (2021) who provide a framework of common sources of uncertainty in the general context of data analyses. We thereby focus on the four epistemic sources of uncertainty resulting from a lack of knowledge about the right strategy to *analyze* the data, namely, method uncertainty, model uncertainty, data preprocessing uncertainty, and parameter uncertainty. We translate this general framework to the context of GSA to outline the choices a researcher is confronted with. See Table 1 for an overview. Thereby, we assume that the data generation has been completed and the (raw) gene expression data set is available. Note that there are additional sources of uncertainty anchored in the *generation* of the gene expression data set that can also lead to variability in the results even if the analysis strategy is fixed. While we do not include these sources of uncertainty in our analysis, we address them briefly in Section 1 in the Supporting Information.

**TABLE 1:** Overview of the sources of uncertainty arising in the analysis of the gene expression data using gene set analysis.

Uncertainty source	Description	Example
Model uncertainty	Which model describes the underlying system best?	Should I choose an ORA or an FCS method?
Method uncertainty	Which method should I choose?	Should I choose GSEA or DAVID?
Data preprocessing uncertainty	Which approach should I choose to generate the input object required by the GSA method?	Which approach to prefiltering should I choose?
Parameter uncertainty	Which values of the input parameter for the GSA method should I choose?	Should I choose Gene Ontology or KEGG as the gene set database?

In the context of GSA, *method uncertainty* refers to the uncertainty about the choice of a method to investigate differential enrichment of the gene sets between the conditions. The wide variety of available methods from which a researcher has to choose becomes clear when inspecting the comprehensive reference database on GSA methods by Xie, Jauhari, and Mora (2021). This database contains around 150 GSA methods assigned to Overrepresentation Analysis (ORA) and Functional Class Scoring (FCS) alone. However, there is little guidance on how to make a suitable choice. Ballouz, Pavlidis, and Gillis (2017) claim that there is even no general consensus on how to benchmark the available methods to derive such guidance. Furthermore, Xie, Jauhari, and Mora (2021) make a threefold observation. First, each benchmark study typically compares only a small subset of all available methods, meaning that the performance of the majority of methods has not even been investigated beyond the original papers that introduced them. Second, the benchmark studies often contradict each other in their results regarding the best and poorest performers, resulting in some methods simultaneously occupying the top and bottom positions in different performance rankings. Lastly, there appears to be a discrepancy between the performance of these methods and their popularity among the users. These observations underline that the right choice of a GSA method is far from clear in practice.

Of important note, we use the general term *method* to refer to both theoretical and computational methods. By *theoretical* method, we mean the method's general concept and features as typically described textually in an original scientific article. In contrast, we refer to their practical implementations in the form of web‐based applications or software packages as *computational* methods (and use typewriter font for their corresponding name, that is, “method”). For instance, the *theoretical* method “Gene Set Enrichment Analysis” from the publication of Subramanian et al. (2005) is implemented in several *computational* methods. While the computational method GSEA (Mootha et al. 2003; Subramanian et al. 2005), which is a web‐based application, exactly implements Gene Set Enrichment Analysis as introduced in the original paper, the user can also choose from computational methods that implement variations of it, such as GSEAPreranked and GSEA provided by the R package clusterProfiler (Wu et al. 2021). In the remainder of this paper, we will use the generic terms “method” and “method uncertainty” and not further address the distinction between theoretical and computational methods.

In the context of GSA, *model uncertainty*, which arises from the uncertainty about how to adequately model the underlying system, is implicitly included in method uncertainty. Imagine, for instance, that a researcher chooses between the two popular methods DAVID and Gene set Enrichment Analysis. This implies the choice between the general approaches ORA and FCS and their assumptions on the underlying biological system on which the corresponding methods are based. For instance, while ORA methods typically assume a hypergeometric distribution as the underlying null distribution, FCS methods assess differential enrichment nonparametrically.

No less pronounced than method uncertainty (and the implied model uncertainty) is the uncertainty about how to process the gene expression data into the format required by the chosen method (*data preprocessing uncertainty*). In earlier work, we have observed that this aspect is often neglected in practical applications of GSA as well as user manuals provided alongside the corresponding methods (Wünsch et al. 2023). This, again, results in little guidance for researchers. One of many examples is the choice of a method for differential expression analysis to generate the required input for ORA methods, for which the popular methods limma (Law et al. 2014), DESeq2 (Love, Huber, and Anders 2014), and edgeR (Robinson, McCarthy, and Smyth 2010) are only a small selection of all available options.

Finally, the researcher is confronted with uncertainty about the choice of parameter values within the chosen GSA method (*parameter uncertainty*), arising from existing flexibility in the parameters to adapt the analysis strategy to the given research question. An example is the parameter “gene set database,” for which there are a variety of options that differ in structure and additional aspects related to the modelling of the underlying biological system. Note that a loose form of guidance is available for some parameters in the form of default values, while for others, such as the gene set database, the user has to make the decision autonomously.

Combined with a lack of clear practical guidance, these uncertainties might impel users to select the GSA method, its underlying parameters (including parameters such as the gene set database), and data preprocessing approach based on which choice(s) yield(s) preferable results to their research question. Such practice may seem natural at first glance. After all, pitfalls of analysis strategies often only come to light when the analyses are run, and it is then acceptable to modify the original planned strategy. Some researchers may not realize that choosing the method that yields preferable results is more than just a reaction to unforeseen problems of the planned analysis strategy. It indeed amounts to the *questionable research practice* termed *cherry‐picking*.

Hoffmann et al. (2021) accentuate that a selective reporting of research findings generated using this “cherry‐picking approach” often results in presenting overoptimistic and thus nonreplicable findings including false positive test results and inflated effect sizes, as outlined in the introduction. Assuming the well‐intentioned nature of researchers engaging in cherry‐picking (as opposed to someone who maliciously intends to manipulate the results), we want to realistically assess the extent to which their tweaking of the GSA results in the above‐described manner leads to overoptimism.

## 3 Design of the Study

**FIGURE 1:**
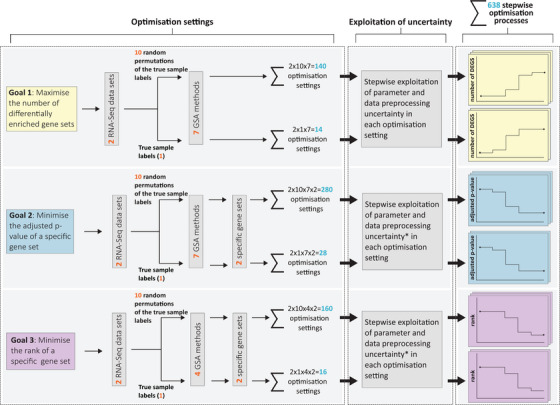
Overview of the study design to investigate the potential for overoptimistic results in a total of 638 optimization settings, resulting in 638 separate optimization processes. The asterisk “*” refers to the fact that for goals 2 and 3, the choice gene set database cannot be exploited in the corresponding optimization processes.

The study aims to systematically assess the potential of GSA for the generation of overoptimistic and thus nonreplicable results as a consequence of the exploitation of the inherent uncertainties combined with selective reporting. We expect the potential of overoptimism to vary depending on the considered GSA method, gene expression data set, and goal of the analysis. Therefore, we imitate the behavior of a hypothetical researcher in their attempt to tweak (i.e., to *optimize*) the GSA results in a variety of settings described in Section 3.1. The exploitation of uncertainty in each fictive setting leads to a separate *optimization process*. While the details of the uncertainty exploited are presented in Section 3.2, the structure underlying each optimization process with its different steps is described in Section 3.3. For a graphical illustration of the overall study design, see Figure 1.

### 3.1 Settings

Each setting (also referred to as *optimization setting*) is defined through a unique combination of

(i) one (of three) optimization goal(s) that drive(s) the optimization,
(ii) one (of two) gene expression data set(s),
(iii) one specific assignment of the conditions to the samples (either the true sample labels or one of the ten random permutations),
(iv) one (of seven) GSA method(s),
(v) for two of the goals mentioned in (i): one (of two) gene set(s).

This results in a total of 638 optimization settings and correspondingly 638 optimization processes. For each optimization process, overoptimism in the tweaked results is assessed relative to the “default” results, that is, the results arising from the default choice in all analysis steps of GSA in which the hypothetical researcher faces uncertainty. For a description of the specification of the default choices, see Section 3.3. In the remainder of this section, we elaborate on aspects (i)–(v) that define the individual optimization settings.

***(i) Optimization goals***

When exploiting the uncertainty inherent to GSA in an attempt to tweak the results, a researcher typically has a specific criterion (i.e., “goal”) in mind. In the following, we define three (distinct) possible goals, resulting in three different ways the hypothetical researcher tries to induce results that they consider satisfactory.

First, it can be assumed that a researcher would not undergo the complex and laborious procedure of generating a gene expression data set and analyzing it without hoping for significant results in the first place. Furthermore, a large number of significantly enriched gene sets provides flexibility when reporting a study. One may focus on those significantly enriched gene sets that better fit the “storyline” of the paper. In the framework of our study, we translate this preference for a large number of significantly enriched gene sets by formulating optimization goal 1 as “maximizing the number of differentially enriched gene sets.”

The two remaining goals follow an alternative intuition. Researchers often have an explicit expectation as to which gene set constitutes an interesting research finding and therefore try to maximize its relevance in the GSA results. Relevance of a specific gene set may be defined in different ways, leading us to consider the optimization goals 2 and 3: “minimizing its adjusted p‐value” and “minimizing its rank among the remaining gene sets,” respectively. We thereby assess the extent to which a user can influence the ranking of the gene sets in the GSA results to induce a significant association between the condition of interest and a particular gene set. In the context of our study mimicking a hypothetical researcher's approach, we have to choose the gene sets to be involved in goals 2 and 3, see below (v).

***(ii) Gene expression data sets***

The three goals are separately considered for two RNA‐Seq gene expression data sets. The selection criteria for the data sets, leading to two real data sets frequently used for benchmarking, can be found in Section 3.1 in the Supporting Information. The first data set, in the following referred to as “Pickrell data set,” contains gene expression measurements of 52,580 genes that were extracted from the lymphoblastoid cell lines of 69 independent Nigerian individuals (Pickrell et al. 2010). The samples are labeled according to the sex of the individuals (*n* = 29 males, *n* = 40 females). We obtained the data set from version 1.34.0 of the R package TweeDEseqCountData (Gonzalez and Esnaola 2022).

The second data set, referred to as “Bottomly data set,” was used to detect genes that are differentially expressed between the two inbred mouse strains “C57BL/6J” (*n* = 10) and “DBA/2J” (*n* = 11) from a total of 36,536 genes (Bottomly et al. 2011). We obtained this data set from the ReCount project (Frazee, Langmead, and Leek 2011).

***(iii) Sample labels***

In our study, we focus on scenarios where the ground truth is that no gene sets are differentially enriched between the conditions of interest. Any improvement of the GSA results through the exploitation of uncertainty (in the respective contexts of goals 1–3) can thus be interpreted as overoptimism. Given the above‐described gene expression data sets, we obtain such scenarios by permuting the true sample labels randomly across the samples, thereby removing the biological meaningfulness from the data. We repeat the permutation procedure 10 times, resulting in 10 random permutations of the true sample labels.

However, in reality, overoptimism might also occur when the ground truth is truly unknown. Researchers might still aim to improve the results, for instance, to obtain a better storyline of the gene sets to report as findings. We therefore repeat our study on the true (i.e., nonpermuted) sample labels. Note, however, that we cannot interpret the corresponding improvement as overoptimism exclusively. It may also be possible that the default analytical choice(s) did not model the underlying biological system adequately—and that the “optimized” choice of the analysis strategy (parameter setting, data preprocessing approach) happens to better do so. When considering data sets with the true sample labels, our focus is therefore on the quantification of the *variability* in the GSA results as the consequence of parameter and data preprocessing uncertainty, rather than on overoptimism.

***(iv) GSA methods***

We consider a selection of seven GSA methods from the reference database provided by Xie, Jauhari, and Mora (2021). Six of these seven methods are chosen for their popularity, whereas the seventh is selected for its good overall performance. Note that this selection procedure results in a restriction to methods categorized as ORA or FCS. For further details on the selection process and short descriptions of the resulting GSA methods, inspect our earlier work (Wünsch et al. 2023). An overview of the methods included in our study can be found in Table 2.

**TABLE 2:** Overview of the GSA methods included in our study.

GSA method	Implemented in	Selection criterion	Introduced by
GOSeq	R	Popularity	Young et al. (2010)
DAVID	Web	Popularity	Huang, Sherman, and Lempicki (2009a, 2009b)
ORA by clusterProfiler	R	Popularity	Wu et al. (2021)
PADOG	R	Performance	Tarca, Bhatti, and Romero (2013)
GSEA by clusterProfiler	R	Popularity	Wu et al. (2021)
GSEA	Web	Popularity	Subramanian et al. (2005), Mootha et al. (2003)
GSEAPreranked	Web	Popularity	Subramanian et al. (2005), Mootha et al. (2003)

It is important to note that while the choice of a GSA method (and the underlying model) is a source of uncertainty in practice, we do not exploit method uncertainty in the individual optimization processes in our study. Instead, the optimization for a given optimization goal, gene expression data set, and assignment of the sample labels is performed for each GSA method separately: in each optimization process, the GSA method is considered to be fixed. This has the advantage that we can better investigate and compare the behaviors of the methods. Method uncertainty is, however, implicitly considered when comparing the results of the optimization processes across the GSA methods. Of additional note is that three methods from our selection, namely, DAVID, GSEA, and GSEAPreranked, are web‐based applications for which all optimization processes have to be performed manually (i.e., by hand). Time constraints lead us to limit our study for these three methods to the optimization goals 1 and 2, whereas goal 3 is omitted.

***(v) Gene sets (for goals 2 and 3)***

Goals 2 and 3 refer to a specific gene set whose adjusted p‐value or rank, respectively, is to be minimized within the optimization process. In practice, the preferences of researchers for a specific gene set arise from previous experiences, literature, or the hypotheses they want to investigate. In the context of our study, we have to define the preferences of our hypothetical researcher in an arbitrary but plausible way. In our initial attempt to select two common gene sets for all GSA methods, we encountered obstacles that led us to consider different gene sets for the different methods. A description of the selection process alongside an overview of the selected gene sets for all methods and both gene expression data sets is provided in Section 3.1 in the Supporting Information. For simplicity, we only refer to “gene set 1” and “gene set 2” in the rest of this paper.

### 3.2 Exploited Uncertainties

**FIGURE 2:**
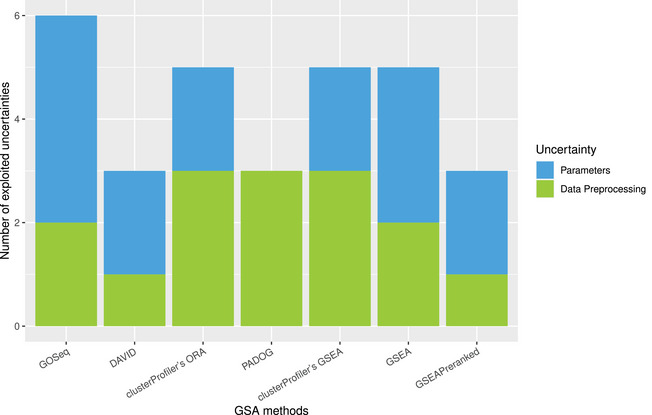
Overview of the number of choices affected by uncertainty that are exploited in our study for each method under investigation. The numbers are additionally split based on the type of uncertainty the corresponding choice is associated with.

An overview of the numbers of exploited data preprocessing and parameter uncertainties for each of the seven investigated GSA methods can be found in Figure 2. This figure illustrates that the ratio between data preprocessing and parameter uncertainties can differ notably between the methods. For instance, PADOG does not offer any flexibility in terms of the parameter setting, while for GOSeq, four of the six exploited uncertainties arise from parameter uncertainty. Our underlying assumption that researchers are *well intentioned* implies that we deliberately renounce to exploit some uncertainties when we consider that this would amount to a willful manipulation of the results. This concerns adaptions in the GSA workflow that might be inappropriate in the given statistical or biological context or those explicitly discouraged by the author of the respective method. Furthermore, we only consider those adaptions that can be carried out without excessive effort.

***Data preprocessing uncertainty***

We exploit the uncertainty about the approach to prefiltering of lowly expressed genes, the removal of duplicated gene IDs as a result of gene ID conversion (while we do not exploit the choice of an approach to gene ID conversion itself), the method for differential expression analysis (“DE method”) for ORA and some of the FCS methods, and the method for transformation (which typically includes normalization) for the remaining FCS methods. Note that not all of these steps apply to each of the GSA methods from our selection because the methods often differ in the required input object. Furthermore, the options available for one step might depend on the choice made in a previous step. For instance, the approach to prefiltering typically differs between different DE methods. For a more detailed description of the exploited uncertainties in the data preprocessing steps, including a description of the respective options, see Section 2.1 in the Supporting Information.

***Parameter uncertainty***

In the process of optimizing the results, we exploit uncertainty about the choice of the gene set database, the so‐called “universe” (for ORA), the method for the calculation of the p‐value, as well as the gene‐level statistic and the weight for FCS. Note that not all uncertainties apply to all GSA methods. For a more detailed overview, refer to Section 2.2 in the Supporting Information. Note that we exploit the choice of the gene set database only for goal 1, that is, when maximizing the number of differentially enriched gene sets. Indeed, the specific gene sets considered for goals 2 and 3, stemming from a certain gene set database, typically do not exist in the same form in another gene set database. The reason for this is that the gene set databases can differ greatly in their structure and the gene sets they contain. Furthermore, for goal 1, we restrict ourselves to the gene set databases offered alongside the individual GSA methods and do not work with a customized gene set database that can be uploaded to the method.

### 3.3 Stepwise Optimization Process

In this section, we focus on the structure underlying all optimization processes of the GSA results. Real researchers, particularly if well intentioned, are unlikely to try out all combinations of analytical choices affected by uncertainty. Instead, they are more likely to tweak the results in a stepwise manner—consciously or subconsciously. We also adopt a stepwise approach in our optimization processes. This means that we exploit the uncertainties in a specific order such that the optimal choice of a specific step (in the context of the corresponding optimization goal) is based on the optimal choices from the previous step(s). Optimization is thus not performed globally for all uncertainties simultaneously, because we consider such an approach unrealistic in practice.

More precisely, we specify the order of the choices a priori and (roughly) in alignment with the natural order of the corresponding steps required to conduct GSA. For instance, uncertainties arising from data preprocessing uncertainty are exploited before addressing those emerging from parameter uncertainty. The intuition behind this order is that a user of GSA must perform data preprocessing before running the actual GSA method (and specifying the corresponding parameters). However, there are instances where we have to make exceptions to this order, namely, when it leads to practical difficulties in the optimization processes. For example, for ORA methods, the choice of the DE method is optimized before prefiltering (while the natural order is reversed). The reason for this is the previously mentioned fact that different DE methods propose different prefiltering approaches.

For each uncertain choice in the GSA workflow, we set a default option a priori. For those steps where a common default option exists (which is often the case for parameters), we set the default choice for our study accordingly. For those steps for which no default exists (such as the gene set database for many methods), we set the default choice for our analysis arbitrarily. Furthermore, we specify an ordering of the alternative options, which will be used as a criterion in case several alternative options yield exactly the same improvement; see below.

Having specified the set of uncertain choices (i.e., steps), their order, the default, and valid alternative options including their order, the optimization process proceeds as follows. The optimization process starts with all choices in their default configuration (step 1). The value to be optimized (i.e., the number of differentially enriched gene sets for goal 1, the adjusted p‐value, or rank of the considered gene set for goals 2 and 3, respectively) obtained in this default configuration is reported as the starting point (*default results*). Then, in step 2, the first uncertain choice is exploited such that the default results are compared to all results stemming from the alternative options. An alternative option is then adopted as the *optimal option* for this step if it leads to an improvement of the results, namely, an increased number of differentially enriched gene sets (for goal 1), a decreased adjusted p‐value of the considered gene set (for goal 2), or a decreased rank of the considered gene set among the remaining ones (for goal 3). If several alternatives lead to an improvement of the GSA results, the alternative that leads to the greatest improvement is selected. If the improvements are equally strong, the alternative option is selected according to the preliminary fixed ordering. In contrast, the default option for this step is retained if none of the alternative options leads to an improvement. The GSA results arising from the optimal option are then denoted as the *current optimal results*.

In the third step, the procedure just described is repeated for the second uncertain choice. Thereby, the current optimal results from step 2 serve as the default results in this step. This procedure is carried out in the same manner for each uncertain step and in the order established previously. That way, the optimal choice in each step is based on the optimal choices and corresponding optimal results from all previous steps.

By examining the results optimized in this stepwise manner and comparing them to the respective default results, it is possible to assess the variability in the results generated by exploiting uncertainty. Most importantly, for the permuted sample labels, it is possible to assess the level of overoptimism induced by this optimization process.

## 4 Results

The presentation of the results of our study is structured according to the three optimization goals. For each goal, the results are further split according to the assignment of the sample labels (10 permutations versus true sample labels). For each method considered for the respective goal, we then contrast the “tweaked” GSA results from the associated optimization processes to the corresponding “default” results. This allows for a comparison between the GSA methods regarding the potential for overoptimism.

Since the results are generally similar for the Pickrell and the Bottomly data set, we focus on the results for the former data set, while referring to Section 4.2 the Supporting Information for the results of the Bottomly data set. To gain a better understanding of the stepwise structure of the optimization processes using a concrete example, see Section 4.1 in the Supporting Information.

For simplified readability, we abbreviate the term “sample label permutation” with “permutation” in the following.

### 4.1 Results for Goal 1: Maximize Number of Differentially Enriched Gene Sets (DEGS)

**FIGURE 3:**
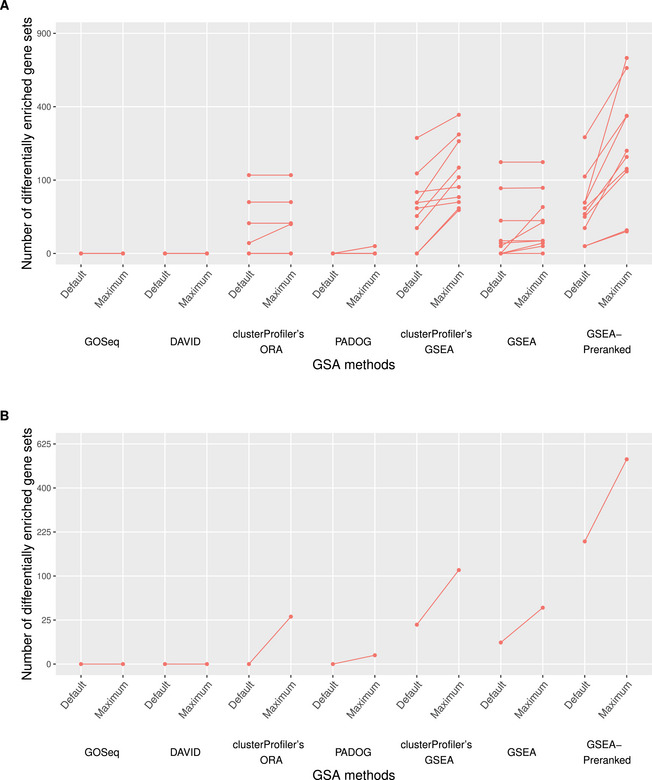
Goal 1: The optimized numbers of differentially enriched gene sets in the Pickrell data set (“Maximum”), obtained through the exploitation of uncertainty, are compared to the corresponding numbers resulting from the default analytical choices (“Default”). For each optimization process, the associated optimized and the default number are connected through a line. (A) presents the results for the 10 random permutations and (B) for the true sample labels. On the x‐axis, the individual methods investigated in the context of goal 1 are displayed. Special attention must be paid to the transformed scale of the y‐axis. This transformation enables visibility of small increases in the number of DEGS, while particularly large increases appear smaller than they actually are.

For a graphical illustration of the results for goal 1, see figure 3.

***Random sample label permutations:***

Overoptimism concerning goal 1 mainly affects the GSEA‐based methods (GSEA, GSEAPreranked, and clusterProfiler's GSEA). In particular, the number of DEGS cannot be increased for any of the permutations for GOSeq and DAVID and only in a small minority of permutations for clusterProfiler's ORA and PADOG. For PADOG especially, the number of DEGS does not exceed 1 DEGS after the exploitation of uncertainty in any of the permutations.

For the web‐based application GSEA, an increase in the number of DEGS is obtained for just over half of the permutations, in three of which the initial number of 0 can be tweaked to a nonzero one. For instance, we observe an increase from 0 to 40 DEGS in one permutation solely through the specification of an alternative weighting pattern of the genes in the computation of the enrichment score. Note that for the web‐based method GSEA, the set of analytical choices leading to a tweak in the corresponding results differs greatly between the permutations.

The observations for the remaining (GSEA‐based) methods GSEAPreranked and clusterProfiler's GSEA are particularly striking. Even before exploiting any uncertainty, the initial numbers of DEGS considerably exceed 0 in the vast majority of permutations. Note that this observation cannot be viewed as overoptimism in the sense considered in this paper. However, it provides information about the general reliability of these GSA methods. In particular, it coincides with the observation that FCS methods that require as input an already ranked list of the genes (as opposed to generating the ranking internally) have inflated false discovery rates (Maleki et al. 2020; Wu and Smyth 2012).

Furthermore, an increase in the number of DEGS can be observed with these two methods for all permutations, amounting to several magnitudes in the great majority of cases. Thereby, a striking pattern as to which analytical choices in the data preprocessing and parameters trigger an increased number of DEGS is particularly visible for GSEAPreranked. Consisting of the choice of the DE method and the assignment of equal weight to all genes in the computation of the enrichment score, the increase from 12 to 196 DEGS in one permutation is only one of many examples where it leads to overoptimistic results.

According to the user manual provided alongside GSEAPreranked, the option of assigning equal weight to each gene in the computation of the enrichment score can be viewed as a “conservative scoring approach.” It is recommended over the default of weighting each gene by its absolute value of the gene‐level statistic when unsure about the biological meaningfulness of the magnitude of the ranking metric for the user's research question (Mootha et al. 2003; Subramanian et al. 2005). A user could thus easily justify the exploitation of this uncertainty with the recommendations from the user manual.

For clusterProfiler's GSEA, the set of uncertain choices leading to the highest increase in the number of DEGS varies more strongly between the permutations. Nevertheless, we also observe notable increases for this method, such as from 0 to 38 DEGS through the choice of the DE method and the prefiltering approach.

***True sample labels:***

For the true sample labels, an increase in the number of DEGS by exploiting data preprocessing and parameter uncertainty is achieved for all GSA methods apart from GOSeq and DAVID. For the latter two, the number of DEGS amounts to 0 before and after the exploitation of uncertainty. For PADOG, a moderate increase from 0 to 1 DEGS is achieved through the choice of prefiltering. In contrast, for GSEAPreranked, the initial number of DEGS, amounting to almost 200, is already exceptionally high and can even be further doubled through the choice of the DE method as part of data preprocessing. This choice proves to be a trigger for increase not only for GSEAPreranked. While for clusterProfiler's ORA, the number of DEGS is thus increased from 0 to 26, the initial number of 20 DEGS is further raised by factor six through the modification of the DE method for clusterProfiler's GSEA.

### 4.2 Results for Goal 2: Minimize Adjusted p‐Value of a Specific Gene Set

**FIGURE 4:**
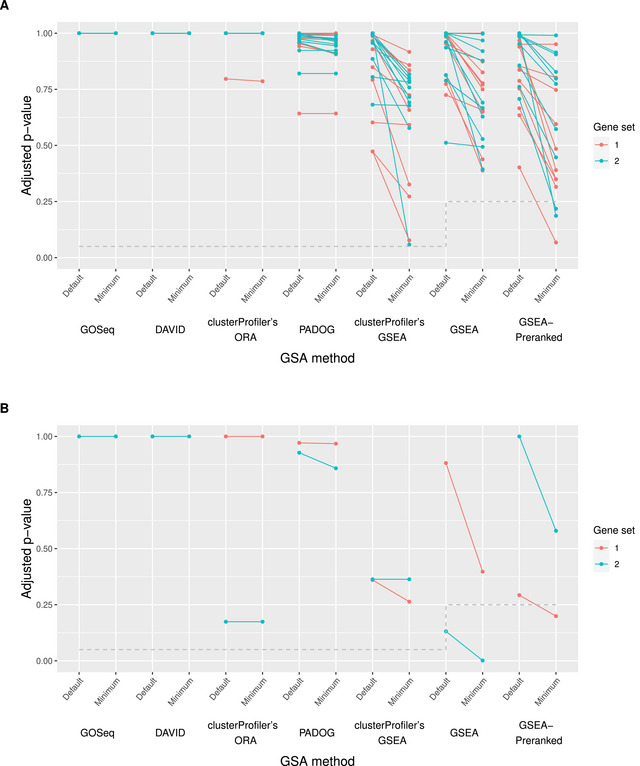
Goal 2: The optimized adjusted p‐values (/q‐values) in the Pickrell data set (“Minimum”), obtained through the exploitation of uncertainty, are compared to the corresponding values resulting from the default analytical choices (“Default”). Note that for the web‐based applications GSEA and GSEAPreranked, the q‐value is used to assess differential enrichment instead of the adjusted p‐value. For each optimization process, the associated optimized and the default adjusted p‐value (/q‐value) are connected through a line. (A) presents the results for the 10 random permutations and (B) for the true sample labels. On the x‐axis, the individual methods investigated in the context of goal 2 are displayed. The results for gene set 1 are shown in red and those for gene set 2 in blue. The dashed gray line indicates the significance threshold for each method below which a gene set is considered differentially enriched.

Note that the significance threshold considered by the web‐based applications GSEA and GSEAPreranked is a *q*‐value of <0.25; see Storey (2002) for a definition of the q‐value. The remaining methods detect a gene set as differentially enriched if its p‐value adjusted using the Benjamini–Hochberg procedure is lower than 0.05. We stick to these default settings in our interpretation of the analyses of goal 2. See Figure 4 for a graphical illustration of the results for goal 2.

***Random sample‐label permutations:***

Analogous to goal 1, the degree of overoptimism regarding goal 2 is the highest for the GSEA‐based methods. Before and especially after exploiting uncertainty, these three methods generally indicate lower adjusted p‐values (/q‐values), respectively, compared to the remaining methods.

Similar to goal 1, the adjusted p‐values of the respective gene sets cannot be tweaked for GOSeq and DAVID (all adjusted p‐values are equal to 1 even after optimization). Similar observations are made for clusterProfiler's ORA and PADOG, where decreases in the adjusted p‐value through the exploitation of data preprocessing and parameter uncertainty are, if at all existent, negligible (i.e., less than 0.02 in the vast majority of permutations).

For the web‐based application GSEA, we observe moderate to notable decreases in the q‐value in the vast majority of permutations. However, none of the optimizations applied in the analysis of the permuted data turns an initially nonsignificant q‐value into a significant one.

In contrast, there are three permutations for GSEAPreranked in which the tweaking of the GSA results leads to the detection of differential enrichment of the respective gene set that was initially not found to be enriched. In particular, we observe a q‐value decrease from 1 to 0.19 in one permutation. As can also be observed in the majority of the remaining permutations, this strong decrease is triggered by the choice of the DE method and the modification of the weighting pattern of the genes in the computation of the enrichment score.

For clusterProfiler's GSEA, we also observe the just‐described set of analytical choices to trigger a decrease in the adjusted p‐value of the respective gene sets in the majority of permutations. Note that we additionally observe the approach to the removal of duplicated gene IDs to be a common trigger of decrease. However, the corresponding effect is always negligible. The strongest decreases using clusterProfiler's GSEA are observed in one permutation for both gene sets, each. They lead from 0.47 to 0.077 and from 1 to 0.058, respectively, such that the corresponding “tweaked” adjusted p‐values remain just above the significance threshold.

***True sample labels:***

Similar to goal 1, a reduction of the adjusted p‐value cannot be achieved for GOSeq, DAVID, and clusterProfiler's ORA for any of the gene sets. In contrast, for GSEAPreranked, an initially nonsignificant q‐value of 0.29 is reduced to the significant value of 0.20 through the change of the DE method in data preprocessing. For the web‐based application GSEA, the initial q‐value of one of the considered gene sets is 0.13, indicating significant differential enrichment even before the exploitation of uncertainty. The modification of the weighting pattern of the genes in the computation of the enrichment score leads to a further decrease to a q‐value of 0.001.

### 4.3 Results for Goal 3: Minimize Rank of a Specific Gene Set

**FIGURE 5:**
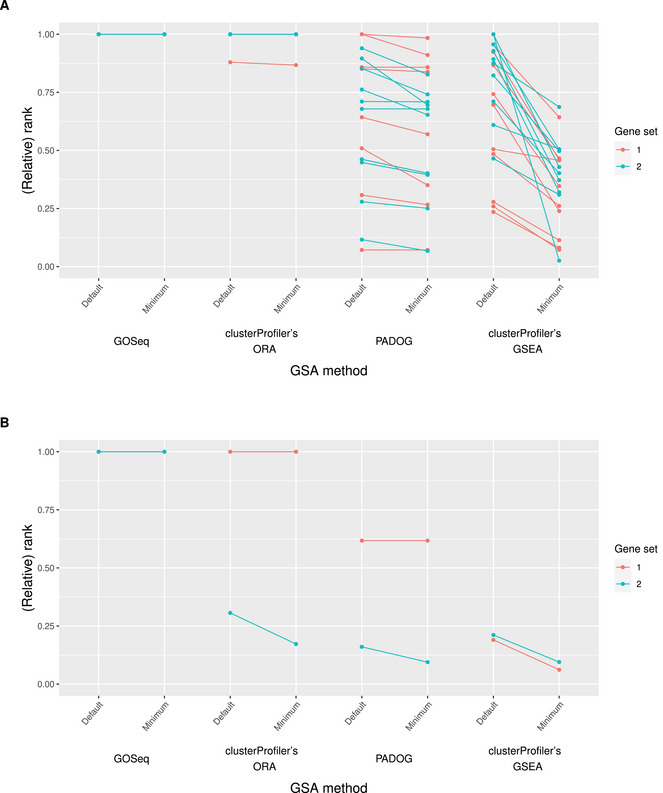
Goal 3: The (relative) ranks in the Pickrell data set (“Minimum”), obtained through the exploitation of uncertainty, are compared to the corresponding values resulting from the default analytical choices (“Default”). For each optimization process, the associated optimized and the default rank are connected through a line. (A) presents the results for the ten random permutations and (B) for the true sample labels. On the x‐axis, the individual methods investigated in the context of goal 3 are displayed. The results for gene set 1 are shown in red and those for gene set 2 in blue.

In our study, we define the rank of a gene set in the GSA results based on the listing of all gene sets that typically stems from the order of their adjusted p‐values. Thereby, the gene set with the lowest adjusted p‐value (indicating the strongest association with the condition of interest) occupies the first position. If several gene sets have an identical adjusted p‐value, we assign them the same rank. Furthermore, we take into account that the number of contained gene sets often varies between GSA results tables, particularly when generated using different GSA methods that often refer to distinct versions of the corresponding gene set database. To ensure comparability between the rank of a gene set across GSA results tables, we therefore divide each rank in the given GSA results table by the maximum assigned rank from that table. The resulting *relative* ranks range from 0 to 1. Thereby, a lower relative rank indicates that the corresponding gene set's adjusted p‐value is generally lower compared to the remaining genes (and vice versa). In particular, an adjusted p‐value of 1 automatically results in a relative rank of 1. In the following interpretation of the analyses of goal 3, we use the expressions “rank” and “relative rank” interchangeably. A graphical illustration of the results is provided in Figure 5.

***Random sample‐label permutations:***

For GOSeq and clusterProfiler's ORA, we observe the same pattern as with goals 1 and 2, namely, that the ranks of the two considered gene sets remain at the highest possible value of 1 before and after the exploitation of uncertainties.

In contrast, for PADOG, we observe many slight to moderate decreases in the relative ranks for the majority of permutations, triggered through the choice of the prefiltering approach or the method to transform the RNA‐Seq data. Note that the relative ranks are generally low even before exploiting any uncertainty, especially compared to the adjusted p‐values from goal 2; see Figure 4. For instance, with all uncertain choices in their default, gene set 2 has a relative rank of 0.12 in one permutation (whereas the corresponding adjusted p‐value amounts to 0.82). This relative rank is further decreased to 0.07 through the choice of the method to transform the RNA‐Seq data, whereas the adjusted p‐value increases to 0.92. This indicates that while the initial adjusted p‐values of all gene sets are already generally high, the adjusted p‐values of the majority of the remaining gene sets increase even more strongly through the exploitation of uncertainties than the adjusted p‐value of gene set 2.

We observe notable decreases in the relative ranks across the permutations for clusterProfiler's GSEA. Analogous to optimization goals 1 and 2, many of these decreases are triggered through the choice of the DE method to generate the required input and the specification of an equal weighting for all genes in the computation of the enrichment score. The observations coincide with the corresponding ones from goal 2, meaning that through the exploitation of the uncertainty, a user does not trigger a decrease in the adjusted p‐value of all gene sets, but specifically modifies the ranking of the gene sets in the GSA results in favor of their “preferred” gene set. For instance, for gene set 2, there is one permutation in which the choice of the DE method and the specification of an equal weight of all genes in the computation of the enrichment score results in a decrease of the relative rank from 1 to 0.026. At the same time, the adjusted p‐value decreases from 1 to 0.058.

***True sample labels:***

For GOSeq, the relative ranks cannot be decreased from their respective initial value of 1 for either of the gene sets. In contrast, for clusterProfiler's ORA, a reduction from 0.31 to 0.17 is triggered through the choice of the universe for gene set 2. For PADOG, the already low relative rank of 0.16 of gene set 2 can be further decreased to 0.09 through the choice of the method for RNA‐Seq transformation. Note, however, that the corresponding adjusted p‐values before and after the exploitation of the uncertainties, respectively, amount to 0.92 and 0.90. This indicates that the adjusted p‐values of the vast majority of remaining gene sets are even closer to 1.

## 5 Discussion

In our analysis, we quantified overoptimism effects resulting from the multiplicity of analysis strategies combined with selective reporting for several popular gene set analysis methods. We thereby considered several types of expectancies/hopes the researchers might have when tweaking their analyses, which we translated into three distinct goals. The maximization of the number of differentially enriched gene sets (goal 1) grants the researcher greater flexibility in generating hypotheses. By minimizing a specific gene set's adjusted p‐value or rank among the remaining gene sets, a researcher attempts to fulfill their expectations as to what constitutes interesting results.

Our study shows that the potential for the generation of overoptimistic results in the context of all three goals particularly affects two methods, namely, GSEA provided by the R package clusterProfiler and the web‐based method GSEAPreranked. Both methods apply variations of Gene Set Enrichment Analysis (Subramanian et al. 2005) commonly known to produce inflated false discovery rates (FDRs) (Wu and Smyth 2012; Maleki et al. 2020). However, we observe their frequent use; see, for example, Reimand et al. (2019) and Lopes‐Ramos et al. (2020). Our study shows that, in addition to the above‐described problem, both methods commonly grant the user to further tweak the results, for example, by changing the weighting pattern of the genes in the assessment of differential enrichment, a modification easily justifiable through information from corresponding user manuals. Since the evidence from our study casts even further doubt on the reliability of thus made research findings, our study should therefore be considered a reinforcement of the recommendation against these methods.

While our work focuses on the exploitation of uncertainty in GSA at the analysis stage, a researcher is in reality already confronted with additional uncertainties during the generation of the gene expression data set. These include (but are not limited to) the choice of the library preparation kit, the quality filter to remove sequences likely to contain errors, and the source of references for gene annotation. A modification in one or more of these aspects can lead to additional variability in the GSA results even when the downstream analysis strategy remains the same. Our focus on later stages of the research process can therefore result in an underestimation of the potential for overoptimistic results. The other way around, the strictly systematic manner of optimizing the results as employed in our work does certainly not fully reflect the reality of data analysis practice, which, in turn, suggests that our study might overestimate overoptimism.

First, the probability of exploiting an existing uncertainty is likely to vary between the different steps, depending on the required effort as well as the researcher's programming expertise and mindset. For example, the adjustment of a preprocessing step, such as changing the method for differential expression analysis when generating the required input for ORA (which requires modifying several lines of code), generally comes with a greater effort compared to changing a parameter value, which can often be done by a single click. Furthermore, the needed efforts are generally higher if the researcher is equipped with little programming experience. The amount of tweaking a real researcher may realistically perform in a practical project is therefore individual. Our selection of analysis steps and options the fictive researcher may choose from does not take this individual variability into account and thus inevitably involves some arbitrariness. This arbitrariness extends to other aspects such as the specification of default options, despite being based on extensive literature research. Most importantly, it may be argued that researchers would not consider as many options in practice.

Second, it is more likely that a real researcher adopts a change in a flexible step if it leads to a noticeable improvement in the results instead of (as is the case in our study design) accepting each improvement, however, small it may be. Indeed, researchers know that each change from the default might require a justification eventually, and may thus be reluctant to engage in changes that do not bring substantial benefits.

Third, we consider the three optimization goals separately, while a real researcher might have several of these goals in mind. For instance, they might want to increase the number of differentially enriched gene sets while, at the same time, trying to reduce the adjusted p‐value of a specific gene set. Likewise, they may have two or more gene sets in mind whose relevance they want to simultaneously increase in the GSA results. On the one hand, considering a single optimization goal as fixed, as in the fashion of our study, might lead to an underestimation of the potential for overoptimistic results since we ignore an additional source of multiplicity. However, pursuing multiple goals simultaneously might make it more difficult to induce satisfactory results in a cherry‐picking manner, such that the resulting overoptimism in the GSA results may actually be less pronounced. In future work, it would therefore be interesting to investigate the effect on the level of overoptimism resulting from the consideration of multiple optimization goals.

Fourthly, in the context of goal 1, there are several optimization processes in which the *default* number of differentially enriched gene sets (i.e., before performing any optimization) is already substantial. It could be increased even further for many of these cases; in reality, however, a researcher would be unlikely to increase an already high number of significant results. While a higher number of differentially enriched gene sets offers a higher flexibility regarding the storyline of the paper and biological understanding of the results, it can also complicate their interpretation and reporting, even forcing the researcher to report only a subset of the detected gene sets. Our study does not take this into account and assumes that the researcher would always be interested in increasing the number of significant gene sets.

To sum up, our study unavoidably requires a certain amount of simplifications, which may not only imply an underestimation of the overoptimistic effect of interest but also its overestimation.

A further aspect of our study that is subject to arbitrariness is the choice of the gene sets whose adjusted p‐value or rank (for optimization goals 2 and 3, respectively) we attempted to minimize. Different choices of gene sets might lead to different extents to which overoptimistic results can be achieved and the gene sets considered in our study only make up a small fraction of the gene sets provided by the gene set database in the individual methods. Furthermore, for goals 2 and 3, our study focused mainly on gene sets provided by the gene set database GO (with subontology “Molecular Function”). It would therefore be interesting to extend the selection of gene sets, additionally considering gene sets from other gene set databases.

To address the arbitrariness in our study design when *mimicking a hypothetical researcher*, it would be interesting to perform a real‐life multianalyst experiment in the spirit of Silberzahn et al. (2018). This would imply recruiting several teams of analysts and presenting them with the same research question and RNA‐Seq data set. The task would then be to investigate the variation in the different steps of the chosen analysis strategies and in the results between the teams. This experiment would additionally allow for an assessment of the meaningfulness of the tweaked GSA results in the context of the conditions of interest. Note that such experiments may also be conducted with students as analysts in the context of undergraduate teaching, see, for example, Heyman and Vanpaemel (2022).

Despite its limitations, our study clearly encourages readers of publications presenting GSA results to interpret them cautiously—that is, with the inherent uncertainties and possible overoptimism in mind. Our study reveals that for some methods, it is relatively easy to cherry‐pick in the context of GSA and that the resulting overoptimism is sometimes substantial. Although we obviously cannot provide evidence of the extent of cherry‐picking in practice (since it happens behind closed doors and is naturally never reported), we conjecture that its incidence is nonnegligible.

What can “real” researchers do to avoid overoptimism when conducting gene set analysis? It is important to not only report those analyses that are identified as “most preferable” *after* inspecting the results. GSA is often performed as part of exploratory research, which makes it difficult to decide on all aspects and details of the analysis before running it. Helpful guidance—may it be in the form of neutral comparison studies supporting the method's choice or user manuals provided alongside the GSA methods—is often scarce, making the prespecification of the analysis strategy even more complex. In this context, it is certainly not realistic to require researchers to fix all analytical choices in advance. A valid alternative approach would be to report the GSA results obtained with several analysis strategies in an effort to transparently disclose and integrate the underlying uncertainties while refraining from reporting only the “best” ones in a cherry‐picking manner. Alternatively, one may select the analysis strategy after running the analyses—and thus identifying potential problematic behaviors of some of the analysis strategies—but *without* looking at the “main results” (which is formalized in terms of “goals” in our study). This latter approach can be seen as a relatively safe compromise between the full prespecification of the analysis strategy, which lacks flexibility in an exploratory setting, and the results‐driven selection of analysis strategies, which often leads to overoptimism as demonstrated through our study.

## Conflicts of Interest

The authors have declared no conflict of interest.

### Open Research Badges

This article has earned an Open Data badge for making publicly available the digitally‐shareable data necessary to reproduce the reported results. The data is available in the Supporting Information section.

This article has earned an open data badge “**Reproducible Research**” for making publicly available the code necessary to reproduce the reported results. The results reported in this article were reproduced partially due to the complexity of the analyses.

## Supporting information

Supporting Information

Supporting Information

## Data Availability

All data and code that support the findings of this study are openly available on GitHub. Code and data to reproduce the study for the R‐based methods are additionally available in the Supporting Information.

## References

[bimj70016-bib-0001] Ballouz, S. , P. Pavlidis , and J. Gillis . 2017. “Using Predictive Specificity to Determine When Gene Set Analysis Is Biologically Meaningful.” Nucleic Acids Research 45, no. 4: e20. 28204549 10.1093/nar/gkw957 PMC5389513

[bimj70016-bib-0002] Bottomly, D. , N. A. Walter , J. E. Hunter , et al. 2011. “Evaluating Gene Expression in C57BL/6J and DBA/2J Mouse Striatum Using RNA‐Seq and Microarrays.” PLoS One 6, no. 3: e17820. 21455293 10.1371/journal.pone.0017820 PMC3063777

[bimj70016-bib-0003] Frazee, A. C. , B. Langmead , and J. T. Leek . 2011. “ReCount: A Multi‐Experiment Resource of Analysis‐Ready RNA‐Seq Gene Count Datasets.” BMC Bioinformatics 12: 1–5. 22087737 10.1186/1471-2105-12-449 PMC3229291

[bimj70016-bib-0004] Gonzalez, J. R. , and M. Esnaola . 2022. tweeDEseqCountData: RNA‐Seq Count Data Employed in the Vignette of the tweeDEseq Package . R package version 1.34.0.

[bimj70016-bib-0005] Held, L. , C. Micheloud , and S. Pawel . 2022. “The Assessment of Replication Success Based on Relative Effect Size.” The Annals of Applied Statistics 16, no. 2: 706–720.

[bimj70016-bib-0006] Heyman, T. , and W. Vanpaemel . 2022. “Multiverse Analyses in the Classroom.” Meta‐Psychology 6: MP.2020.2718.

[bimj70016-bib-0007] Hoffmann, S. , F. Schönbrodt , R. Elsas , R. Wilson , U. Strasser , and A.‐L. Boulesteix . 2021. “The Multiplicity of Analysis Strategies Jeopardizes Replicability: Lessons Learned Across Disciplines.” Royal Society Open Science 8, no. 4: 201925. 33996122 10.1098/rsos.201925 PMC8059606

[bimj70016-bib-0008] Huang, D. W. , B. T. Sherman , and R. A. Lempicki . 2009a. “Bioinformatics Enrichment Tools: Paths Toward the Comprehensive Functional Analysis of Large Gene Lists.” Nucleic Acids Research 37, no. 1: 1–13. 19033363 10.1093/nar/gkn923 PMC2615629

[bimj70016-bib-0009] Huang, D. W. , B. T. Sherman , and R. A. Lempicki . 2009b. “Systematic and Integrative Analysis of Large Gene Lists Using DAVID Bioinformatics Resources.” Nature Protocols 4, no. 1: 44–57. 19131956 10.1038/nprot.2008.211

[bimj70016-bib-0010] Ioannidis, J. P. 2005. “Why Most Published Research Findings Are False.” PLoS Medicine 2, no. 8: e124. 16060722 10.1371/journal.pmed.0020124 PMC1182327

[bimj70016-bib-0011] Law, C. W. , Y. Chen , W. Shi , et al. 2014. “Voom: Precision Weights Unlock Linear Model Analysis Tools for RNA‐Seq Read Counts.” Genome Biology 15, no. 2: 1–17. 10.1186/gb-2014-15-2-r29 PMC4053721 24485249

[bimj70016-bib-0012] Lopes‐Ramos, C. M. , C.‐Y. Chen , M. L. Kuijjer , et al. 2020. “Sex Differences in Gene Expression and Regulatory Networks Across 29 Human Tissues.” Cell Reports 31, no. 12. 10.1016/j.celrep.2020.107795 PMC7898458 32579922

[bimj70016-bib-0013] Love, M. I. , W. Huber , and S. Anders . 2014. “Moderated Estimation of Fold Change and Dispersion for RNA‐Seq Data With DESeq2.” Genome Biology 15: 550. 25516281 10.1186/s13059-014-0550-8 PMC4302049

[bimj70016-bib-0014] Maleki, F. , K. Ovens , D. J. Hogan , et al. 2020. “Gene Set Analysis: Challenges, Opportunities, and Future Research.” Frontiers in Genetics 11: 654. 32695141 10.3389/fgene.2020.00654 PMC7339292

[bimj70016-bib-0015] Maleki, F. , K. L. Ovens , D. J. Hogan , E. Rezaei , A. M. Rosenberg , and A. J. Kusalik . 2019. “Measuring Consistency Among Gene Set Analysis Methods: A Systematic Study.” Journal of Bioinformatics and Computational Biology 17, no. 5: 1940010. 31856670 10.1142/S0219720019400109

[bimj70016-bib-0016] Mootha, V. K. , C. M. Lindgren , K.‐F. Eriksson , et al. 2003. “PGC‐1α‐Responsive Genes Involved in Oxidative Phosphorylation Are Coordinately Downregulated in Human Diabetes.” Nature Genetics 34, no. 3: 267–273. 12808457 10.1038/ng1180

[bimj70016-bib-0017] Nosek, B. A. , and T. M. Errington . 2020. “What Is Replication?.” PLoS Biology 18, no. 3: e3000691. 32218571 10.1371/journal.pbio.3000691 PMC7100931

[bimj70016-bib-0018] Open Science Collaboration . 2012. “An Open, Large‐Scale, Collaborative Effort to Estimate the Reproducibility of Psychological Science.” Perspectives on Psychological Science 7, no. 6: 657–660. 26168127 10.1177/1745691612462588

[bimj70016-bib-0019] Pickrell, J. K. , J. C. Marioni , A. A. Pai , et al. 2010. “Understanding Mechanisms Underlying Human Gene Expression Variation With RNA Sequencing.” Nature 464, no. 7289: 768–772. 20220758 10.1038/nature08872 PMC3089435

[bimj70016-bib-0020] Popper, K. 2005. The Logic of Scientific Discovery. London: Routledge.

[bimj70016-bib-0021] Reimand, J. , R. Isserlin , V. Voisin , et al. 2019. “Pathway Enrichment Analysis and Visualization of Omics Data Using g: Profiler, GSEA, Cytoscape and EnrichmentMap.” Nature Protocols 14, no. 2: 482–517. 30664679 10.1038/s41596-018-0103-9 PMC6607905

[bimj70016-bib-0022] Robinson, M. D. , D. J. McCarthy , and G. K. Smyth . 2010. “edgeR: A Bioconductor Package for Differential Expression Analysis of Digital Gene Expression Data.” Bioinformatics 26, no. 1: 139–140. 19910308 10.1093/bioinformatics/btp616 PMC2796818

[bimj70016-bib-0023] Silberzahn, R. , E. L. Uhlmann , D. P. Martin , et al. 2018. “Many Analysts, One Data Set: Making Transparent How Variations in Analytic Choices Affect Results.” Advances in Methods and Practices in Psychological Science 1, no. 3: 337–356.

[bimj70016-bib-0024] Simmons, J. P. , L. D. Nelson , and U. Simonsohn . 2011. “False‐Positive Psychology: Undisclosed Flexibility in Data Collection and Analysis Allows Presenting Anything as Significant.” Psychological Science 22, no. 11: 1359–1366. 22006061 10.1177/0956797611417632

[bimj70016-bib-0025] Storey, J. D. 2002. “A Direct Approach to False Discovery Rates.” Journal of the Royal Statistical Society Series B: Statistical Methodology 64, no. 3: 479–498.

[bimj70016-bib-0026] Subramanian, A. , P. Tamayo , V. K. Mootha , et al. 2005. “Gene Set Enrichment Analysis: A Knowledge‐Based Approach for Interpreting Genome‐Wide Expression Profiles.” Proceedings of the National Academy of Sciences 102, no. 43: 15545–15550. 10.1073/pnas.0506580102 PMC1239896 16199517

[bimj70016-bib-0027] Tarca, A. L. , G. Bhatti , and R. Romero . 2013. “A Comparison of Gene Set Analysis Methods in Terms of Sensitivity, Prioritization and Specificity.” PloS One 8, no. 11: e79217. 24260172 10.1371/journal.pone.0079217 PMC3829842

[bimj70016-bib-0028] Ullmann, T. , S. Peschel , P. Finger , C. L. Müller , and A.‐L. Boulesteix . 2023. “Overoptimism in Unsupervised Microbiome Analysis: Insights From Network Learning and Clustering.” PLoS Computational Biology 19, no. 1: e1010820. 36608142 10.1371/journal.pcbi.1010820 PMC9873197

[bimj70016-bib-0029] Wu, D. , and G. K. Smyth . 2012. “Camera: A Competitive Gene Set Test Accounting for Inter‐Gene Correlation.” Nucleic Acids Research 40, no. 17: e133–e133. 22638577 10.1093/nar/gks461 PMC3458527

[bimj70016-bib-0030] Wu, T. , E. Hu , S. Xu , et al. 2021. “clusterProfiler 4.0: A Universal Enrichment Tool for Interpreting Omics Data.” The Innovation 2, no. 3: 100141. 34557778 10.1016/j.xinn.2021.100141 PMC8454663

[bimj70016-bib-0031] Wünsch, M. , C. Sauer , P. Callahan , L. C. Hinske , and A.‐L. Boulesteix . 2023. “From RNA‐Sequencing Measurements to the Final Results: A Practical Guide to Navigating the Choices and Uncertainties of Gene Set Analysis.” WIREs Computational Statistics 16, no. 1: e1643.

[bimj70016-bib-0032] Xie, C. , S. Jauhari , and A. Mora . 2021. “Popularity and Performance of Bioinformatics Software: The Case of Gene Set Analysis.” BMC Bioinformatics 22: 191. 33858350 10.1186/s12859-021-04124-5 PMC8050894

[bimj70016-bib-0033] Young, M. D. , M. J. Wakefield , G. K. Smyth , et al. 2010. “Gene Ontology Analysis for RNA‐Seq: Accounting for Selection Bias.” Genome Biology 11: R14. 20132535 10.1186/gb-2010-11-2-r14 PMC2872874

