# Supplementary material for: To Tweak or Not to Tweak. How Exploiting Flexibilities in Gene Set Analysis Leads to Overoptimism
Source: Biom J. 2024 Dec 19;67(1):e70016. doi: 10.1002/bimj.70016 (PMC11656295; doi:10.1002/bimj.70016)
Supplement: Supplementary file 1 — Supporting Information [file BIMJ-67-e70016-s002.zip › OverOptimism_in_GeneSetAnalysis-main/README.pdf]

README

2024-10-22

Repository for code and documentation for analysis on over-optimism in GSA.

This repository allows you to reproduce the results from our over-optimism analysis on four *R*-based and three web-based GSA methods.

Content:

This repository contains instructions on how to

- **reproduce figures based on intermediate results**: based on the available analysis results for methods ("intermediate results"), the figures from the main manuscript are reproduced.
- **reproduce figures from scratch**: for the *R*-based methods, the analysis is performed from scratch while for the web-based methods, the intermediate results are used. Finally, the figures from the manuscript are reproduced.
- **reproduce the analysis for web-based methods *DAVID*, *GSEA*, *GSEAPreranked***: detailed instructions on how to reproduce the intermediate results for the three web-based methods, containing
  - the generation of required input objects in *R*
  - the optimisation performed in the respective web-based method
  - the documentation of results in the form of screenshots and Excel files

The intermediate results for the web-based applications (which are too large for this code supplement) can be accessed at https://github.com/chillemille/OverOptimism_in_GeneSetAnalysis [last accessed: 22 Oct 2024 2:03 pm].

Important notes

Note 1:

Reproducing all results **from scratch** is possible when using

- identical data sets
- identical seed(s)
- identical *R* package versions/ versions of the web applications
- identical gene set database versions

as used in the original experiments. This is straightforward for the *R*-based methods (using renv), however, it may consume a notable amount of memory. For the web-based methods, reproducing the results requires several intermediate steps (see note 4) and is **very** time-consuming (it took several weeks for our analysis); detailed descriptions are provided below.

Note 2:

The *R* results can be reproduced using *R* package *renv* command *renv::restore()*. See https://rstudio.github.io/renv/articles/renv.html [last accessed: 08 Oct 2024 09:40 am] for information on how to use *renv*.

Note: After installing and activating *renv*, there may be packages that are indicated by the renv.lock but have not been installed on your system yet. These packages should be installed using command `renv::install("package")`, where "package" indicates the affected package that needs to be installed.

Note 3:

I produced the results for the three web-based applications (*DAVID*, *GSEA*, and *GSEAPreranked*) over several months, including the part where I (partly) prepared the required input objects in *R*. Unfortunately, I was not aware of reproducible environments such as *renv* and therefore naively proceeded over the months without ensuring exact reproducibility by documenting the current versions of all packages needed in the process. However, I compared **many, but of course, not all** of the *R* outputs generated using *renv* to those I generated at the time and they were very similar (for instance, the rankings required as input to *GSEAPreranked* differed only from the third decimal place).

Note 4:

The web-based applications were performed by hand (and documented with screenshots) using the following three steps:

1. In *R*: Generate input data sets for web application from initial gene expression data
2. For *GSEA* and *GSEAPreranked*: Further pre-processing steps in Excel (for *GSEA* and *GSEAPreranked*)
3. Data is uploaded to the respective web application and optimisations are performed
4. Results of the optimisation processes are entered manually in *R* (scripts in `R/Code_figures`) for the generation of the results graphs

Important: fully reproducing this process would take weeks!

Reproduce figures based on intermediate results

To reproduce a figure from the paper (based on intermediate results), run the corresponding script from folder `R/Code_figures`. The scripts are named after the figure they generate in the manuscript.

Note, while the *R* scripts source the intermediate results from the GSA methods implemented in *R* internally, the results for the web-based applications *GSEA*, *GSEAPreranked*, and *DAVID* were transferred from the corresponding screenshots **by hand** since the optimisation processes could not be run in *R*.

Reproduce figures from scratch

Note this can take several days, depending on the available resources. The following *R* scripts are stored in the folder `R/Run_optimisations`.

1. Run optimisations for *R*-based GSA methods *GOSeq*, *clusterProfiler*'s ORA, *PADOG*, and *clusterProfiler*'s GSEA in the following scripts:

- **Run_task1_optimisations.R**: required for Figures 3 and S2,
- **Run_task2_3_optimisations.R**: required for Figures 4, 5, S3, and S4,

where tasks 1, 2, and 3 are the optimisation tasks based on which the potential for over-optimism is assessed. Both scripts source all required functions and preprocess the initial gene expression data sets internally.

For the remaining, web-based applications *DAVID*, *GSEA*, and *GSEAPreranked*, the optimisations were performed in the corresponding web-based application (i.e., by hand). To generate the results figures from the manuscript, the optimisation results were therefore also transferred manually to the corresponding *R* scripts (`R/Code_figures`). See below for instructions on how to reproduce the optimisation results for the web-based applications.

2. Generate the results figures (folder `R/Code_figures`)

The *R* scripts to generate the results figures are named directly after the figure.

Detailed: reproduce analysis for web-based methods *DAVID*, *GSEA*, *GSEAPreranked*

Note that for the over-optimism analysis of the web applications, we strongly recommend reading the manuscript as it requires several steps, most of which must be performed by hand. These steps are structured according to note 4 above and described in detail in the following. Note that for the web-based applications, only tasks 1 and 2 are pursued:

- task 1: maximise the number of differentially enriched gene sets
- task 2: minimise the adjusted p-value of a specific gene set

DAVID

The web-based application *DAVID* can be accessed via the following link: https://david.ncifcrf.gov/ [last accessed: 08 Oct 2024 09:30 am]. Our analysis was performed with *DAVID* version 6.8 and the DAVID Knowledgebase v2023q3.

1. Generation of input data sets (folder `R/Optimisation_functions/DAVID`) The *R* script to generate the input files for the Pickrell **and** the Bottomly data set is stored in the file **generate_Inputs_DAVID.R**. These input files either contain lists of differentially expressed genes (.txt files "DEGs_... *.txt") or an alternative universe (i.e. list of background genes; .txt files "universe...*_.txt"). Note that input files containing lists of differentially expressed genes can sometimes be empty if **no genes are detected as differentially expressed** by the given method. No differentially expressed genes logically lead to no differentially enriched gene sets, so these empty files **do not** have to be uploaded to the web application and zero differentially enriched gene sets can be concluded automatically.

The generated input data sets are then stored in the folders - `Results/Intermediate_results/DAVID/Pickrell` - `Results/Intermediate_results/DAVID/Bottomly`

and in there, in subfolders that correspond to the given phenotypes (original phenotypes or phenotype permutations 1-10).

Note that for the Pickrell and the Bottomly data set respectively, the input objects for the web application are identical across both tasks 1 and 2 since the individual optimisation steps are (almost) identical.

2. Run DAVID optimisation Access the link https://david.ncifcrf.gov/ [last accessed: 08 Oct 2024 09:30 am]. An input list generated in step 1 can be uploaded by clicking on **Start Analysis** and submitting the input gene list under `Step 1: Enter Gene List`. Select the identifier as **Ensembl_Gene_ID** and set the list type as **Gene List**.

The optimisation steps are the following (see corresponding screenshots in folder `Results/Screenshots_WebApplications` for more detailed illustrations of the optimisation of task 1):

1. Upload default list of differentially expressed genes (generated using DESeq2; gene set database GO (BP)): **DEGs_DESeq2_... .txt** and run DAVID with all parameters in their default configuration
   - **01Load_Default_GeneList.PNG**: DEGs_DESeq2_... .txt is uploaded (**note** configurations for step 2 and step 3 on blue sidebar)
   - **01Param_Default.PNG**: Geneset database is set to "GO_BP_direct" while "Check Defaults" is unchecked
   - **01Results_Default.PNG**: *DAVID* results table is inspected and the number of differentially enriched gene sets is counted (**for optim. task 1**) or the adjusted p-value of the specified gene set is inspected (**for optim. task 2**) (Excel file offers better visuability)
2. Change method to detect differentially expressed genes: upload **DEGs_limma_... .txt**
   - **02Load_limma_GeneList.PNG**: DEGs_limma_... .text is uploaded (note configurations for step 2 and step 3 on blue sidebar)
   - **02Param_limma.PNG**: Geneset database is set to "GO_BP_direct" while "Check Defaults" is unchecked

- **02Results_limma.PNG**: *DAVID* results table is inspected and the number of differentially enriched gene sets is counted (Excel file offers better visibility)

3. **For task 1 only**: change gene set database to KEGG
   - **03Param_KEGG.PNG**: Geneset database is set to "KEGG_PATHWAY" while "Check Defaults" is unchecked
   - **03Results_KEGG.PNG**: DAVID results table is inspected and for number of differentially enriched gene sets is counted (**for optim. task 1**) or the adjusted p-value of the specified gene set is inspected (**for optim. task 2**) (Excel file offers better visibility)
4. Change set of background genes: upload **universe_DESeq2_... .txt** or **universe_limma_... .txt** according to whether step 1 or step 2 leads to better results w.r.t the optimisation task).
   - **04Load_alternativeUniverse.PNG**: the .txt file containing the alternative universe (based on DESeq2 or limma depending on optim. step 2) is uploaded (**note** configurations for step 2 and step 3 on blue sidebar)
   - **04Param_alternativeUniverse.PNG**: the gene set database is specified based on optim. step 3 while "Check defaults" is unchecked
   - **04Results_alternativeUniverse.PNG**: DAVID results table is inspected and for number of differentially enriched gene sets is counted (**for optim. task 1**) or the adjusted p-value of the specified gene set is inspected (**for optim. task 2**) (Excel file offers better visibility)

In each step, you compare the results w.r.t. the given task to the results from the previous step and choose the option yielding better results.

Important: The documentation shows that the number of differentially enriched gene sets could **NOT** be increased for any of the two gene expression data sets and none of the sample labels (neither true nor permuted). Indeed, there was only one case (Bottomly data set, true sample labels) in which there were gene sets with a significant adjusted p-value. There was one more optimisation step for task 1 than for task 2 (step 3: gene set database KEGG; it never led to an increase in the number of differentially enriched gene sets). The remaining optimisation steps were identical between tasks 1 and 2.

We were therefore able to document the optimisation steps for task 2 directly from the documentation for task 1. It (i.e., the Excel files for each optimisation step for task 1) showed us that both gene expression data sets and for all of the sample labels, the adjusted p-value and rank of the respective gene sets.

- **Demethylation** (GO:0070988) and **t Cell mediated immunity** (GO:0002456) for the Pickrell data set,
- **Metabolic Process** (GO:0008152) and **Cellular Process** (GO:0009987) for the Bottomly data set,

could **never** be decreased for their default value of 1.

GSEA (web-based application)

The application can be downloaded from https://www.gsea-msigdb.org/gsea/index.jsp [last accessed: 08 Oct 2024 09:42 am], for which an account must be created. I performed the experiment on versions 4.2.2/4.2.3 (the exact version can be found on the corresponding screenshot).

1. Generation of inputs in *R* (folder `R/Optimisation_functions/GSEA_Web`) For the Pickrell and the Bottomly data set each, you will find the following three *R* scripts:

- **task1_optimisation_GSEAWeb_... .R**: input generation for task 1
- **task2_optimisation_Demethylation_GSEAWeb_... .R**: input generation task 2; gene set **Demethylation**
- **task2_optimisation_tCell_GSEAWeb_... .R**: Input generation task 2; gene set **t Cell mediated immunity**.

The input data generated from each script is stored in folder `Results/Intermediate_results/GSEA_Web/...Raw`. These contain the gene expression measurements as well as the phenotype assignments (which are stored in

subfolder **Phenotypes**).

2. Further preprocessing in Excel These files stored in `Results/Intermediate_results/GSEA_Web/...Raw` require further processing according to https://software.broadinstitute.org/cancer/software/gsea/wiki/index.php/Data_formats [last accessed: 08 Oct 2024 09:43 am]. For this, inspect Sections

- **GCT: Gene Cluster Text file format (*.gct)** for the preprocessing of the gene expression data set in Excel.
- **CLS: Categorical (e.g. tumour vs normal) class file format (*.cls)** for the preprocessing of the corresponding phenotype assignments.

The corresponding preprocessed files are then stored in subfolder `Prep`.

3. Upload data and make optimisations according to task 1 or 2 In the application, the required data sets are uploaded in the tab **Load data**, after which you need to proceed to the tab **Run GSEA**. Further information on the necessary fields to be clicked and filled out can be obtained from the screenshots in folder `Results/Intermediate_results/GSEA_Web`. Note that in each step, the random seed is set to **149**.

Optimisation steps for tasks 1 and 2 (corresponding screenshots are indented; the documentation of the optimisation processes can also be inspected in the respective *R* file as comments):

1. Upload pre-processed gene expression data set and phenotype assignments: upload **exprdat_default_... .gct** and run the method with all parameters in their default configuration
   - **01Param_Default.PNG**: The default parameters are specified, including
     - the gene expression data set exprdat_default_ ... .gct (tab “Expression dataset”)
     - gene set database GO with subontology Biological Processes (tab “Gene sets database”)
     - the exponent “weighted” in the computation of the enrichment score (tab “Enrichment statistic”)
     - the metric “Signal2Noise” for ranking the genes (tab “Metric for ranking genes”)
     - the random seed 149 (tab “Seed for permutation”)
   - **01Results_Default.PNG**: The overview of the results can be inspected. Obtain number of differentially enriched gene sets by adding up the numbers indicated in row “XX gene sets are significant at FDR < 25%” across
     - “Enrichment in phenotype: 0” and
     - “Enrichment in phenotype: 1” → note that the results (and especially **the relevant adjusted p-value**) can also be inspected in the Excel file of the same name
2. Change RNA-Seq transformation method: upload **exprdat_vst_... .gct**
   - **02Param_vst.PNG**: Gene expression data set is changed to exprdat_vst_ ... .gct (see tab “Expression dataset”)
   - **02Results_vst.PNG**: Overview of results for gene expression data set exprdat_vst_ ... .gct
3. Change pre-filtering method: upload **exprdat_vst_filterByExpr_... .gct**
   - **03Param_filterByExpr.PNG**: Gene expression data set is changed to exprdat_filterByExpr_ ... .gct (see tab “Expression dataset”)
   - **03Results_filterByExpr.PNG**: Overview of results for gene expression data set exprdat_filterByExpr_ ... .gct
4. **For task 1 only**: Change gene set database to KEGG (“c2.cp.kegg...”)
   - **04Param_KEGG.PNG**: Change gene set database to KEGG (see tab “Gene sets database”; **for task 1 only**)
   - **04Results_KEGG.PNG**: Overview of results for gene set database to KEGG (**for task 1 only**)
5. Change metric for ranking genes
   - 5.1 change to **tTest**
     - **05Param_tTest.PNG**: Change ranking (gene-level ranking) metric to “tTest” (see tab “Metric for ranking genes”)
     - **05Results_tTest.PNG**: Overview of results for metric to “tTest”
   - 5.2 change to **Diff_of_classes**

  - **05Param__DoC.PNG**: Change ranking (gene-level ranking) metric to "Diff_of_Classes" (see tab "Metric for ranking genes")
  - **05Results__DoC.PNG**: Overview of results for metric "Diff_of_Classes"
6. Change enrichment statistic
   - 6.1 change to **classic**
     - **06Param__exp0.PNG**: Change exponent parameter (in calculation of enrichment score) to "classic" (see tab "Metric for ranking genes")
     - **06Results__exp0.PNG**: Overview of results for parameter "classic"
   - 6.2 change to **weighted__p1.5**
     - **06Param__exp1KOMMA5.PNG**: Change exponent parameter (in calculation of enrichment score) to "weighted_p1.5" (see tab "Metric for ranking genes")
     - **06Results__exp1KOMMA5.PNG**: Overview of results for parameter "weighted_p1.5"
   - 6.3 change to **weighted__p2**
     - **06Param__exp2.PNG**: Change exponent parameter (in calculation of enrichment score) "weighted_p2" (see tab "Metric for ranking genes")
     - **06Results__exp2.PNG**: Overview of results for parameter "weighted_p2"

In each optimisation step, you compare the results w.r.t. the given task to the results from the previous step and choose the option yielding better results.

Important note: the optimisation of GSEA was carried out over several months and by hand. In this period, the application was updated as well as the gene set database GO (with subontology biological process). The versions that were current in the respective optimisation processes can be found in the screenshots containing the name "Param", namely - in the top left corner for the version of the overall web application - in the specified gene set database (tab **Gene Sets database**) for the version of the gene set database

---

GSEAPreranked

GSEAPreranked is a variant of the above-described web-based method GSEA and can therefore be accessed via the same application (download from https://www.gsea-msigdb.org/gsea/index.jsp [last accessed: 08 Oct 2024 09:39 am]). The experiment was performed on versions 4.2.2 and 4.2.3.

1. Generation of inputs (folder `R/Optimisation_functions/GSEAPreranked`) For the Pickrell and the Bottomly data set each, you will find the following *R* scripts:

- **generateInputs__optimisation__GSEAPreranked__task1__... .R**: Input generation for task 1 for the Pickrell and Bottomly data set + additional documentation of the optimisation procedure as comments (documentation could not be placed in separate .txt files since for some optimisation steps, the options depend on the previous step(s)).
- **generateInputs__GSEAPreranked__task2__... .R**: Input generation for task 2 (additionally, the corresponding optimisation documentations are stored in text files in this folder).

Important note: Unlike the "regular" web application GSEA, for GSEAPreranked, users are recommended to convert the gene IDs to "Gene Symbols", with which the analysis is performed, automatically. As a result, no remapping to gene symbols is performed by the method internally so that the tab "Collapse/Remap to gene symbols" should be set to **No__Collapse**.

2. Further preprocessing in Excel **Note** that further preprocessing must be performed in Excel according to section **RNK: Ranked list file format (*.rnk)** in https://software.broadinstitute.org/cancer/software/gsea/wiki/index.php/Data_formats [last accessed: 08 Oct 2024 09:37 am].

3. Upload data and make optimisations according to task 1 or 2 The required data sets are uploaded in the tab **Load data**, after which you need to proceed to the tab **Run GSEAPreranked**. Further

information on the necessary fields to be clicked and filled out can be obtained from the screenshots in folder `Results/Screenshots_WebApplications`. Note that in each step, the random seed is set to **149**.

Optimisation steps for tasks 1 and 2 (corresponding screenshots are indented; the documentation of the optimisation processes can also be inspected in the respective *R* file as comments):

1. Upload default gene ranking: upload **DESeq2__ranking__ ... .rnk**
   - **01Param__Default.PNG**: The default parameters are specified, including
     - the gene ranking DESeq2_ranking_ ... .rnk (tab "Ranked List")
     - gene set database GO with subontology Biological Processes (tab "Gene sets database")
     - the exponent "weighted" in the computation of the enrichment score (tab "Enrichment statistic")
     - the random seed 149 (tab "Seed for permutation")
   - **01Results__Default.PNG**: The overview of the results can be inspected. Obtain number of differentially enriched gene sets by adding up the numbers indicated in row "XX gene sets are significant at FDR < 25%" across
     - "Enrichment in phenotype: 0" and
     - "Enrichment in phenotype: 1" → note that the results (and especially **the relevant adjusted p-value**) can also be inspected in the Excel file of the same name
2. Change method to rank the genes: upload **limma__ranking__ ... .rnk**
   - **02Param__limma.PNG**: change method to rank genes to limma_ranking_... .rnk
   - **02Results__limma.PNG**: overview of results for limma_ranking_... .rnk

3. **For task 1 only**: change gene set database to KEGG ("c2.cp.kegg...")
   - **04Param__KEGG.PNG**: Change gene set database to KEGG (see tab "Gene sets database"; **for task 1 only**)
   - **04Results__KEGG.PNG**: Overview of results for gene set database to KEGG (**for task 1 only**)
4. Change enrichment statistic
   - 4.1 change to **classic**
     - **06Param__exp0.PNG**: Change exponent parameter (in calculation of enrichment score) to "classic" (see tab "Metric for ranking genes")
     - **06Results__exp0.PNG**: Overview of results for parameter "classic"
   - 4.2 change to **weighted__p1.5**
     - **06Param__exp1KOMMA5.PNG**: Change exponent parameter (in calculation of enrichment score) to "weighted_p1.5" (see tab "Metric for ranking genes")
     - **06Results__exp1KOMMA5.PNG**: Overview of results for parameter "weighted_p1.5"
   - 4.3 change to **weighted__p2**
     - **06Param__exp2.PNG**: Change exponent parameter (in calculation of enrichment score) to "weighted_p2" (see tab "Metric for ranking genes")
     - **06Results__exp2.PNG**: Overview of results for parameter "weighted_p2"

Important note: the optimisation of GSEAPreranked was carried out over several months and by hand. In this period, the application was updated as well as the gene set database GO (with subontology biological process). The versions that were current in the respective optimisation processes can be found in the screenshots containing the name "Param", namely - in the top left corner for the version of the overall web application - in the specified gene set database (tab **Gene Sets database**) for the version of the gene set database

The results from these screenshots are then transferred to the corresponding *R* scripts in folder **R/Code__figures**.

In addition to the screenshots, the optimisation processes are documented in the respective *R* scripts as comments for task 1 (**task1__optimisation__GSEAPreranked__ ... .R** in folder `R/Optimisation_functions/GSEAPreranked`) and in .txt files **task2__documentation__GSEAPreranked__ ... .txt** for task 2).
