# Supplementary material for: To Tweak or Not to Tweak. How Exploiting Flexibilities in Gene Set Analysis Leads to Overoptimism
Source: Biom J. 2024 Dec 19;67(1):e70016. doi: 10.1002/bimj.70016 (PMC11656295; doi:10.1002/bimj.70016)
Supplement: Supplementary file 1 — Supporting Information [file BIMJ-67-e70016-s002.zip › OverOptimism_in_GeneSetAnalysis-main/Results/Figures/FigureS1.pdf]

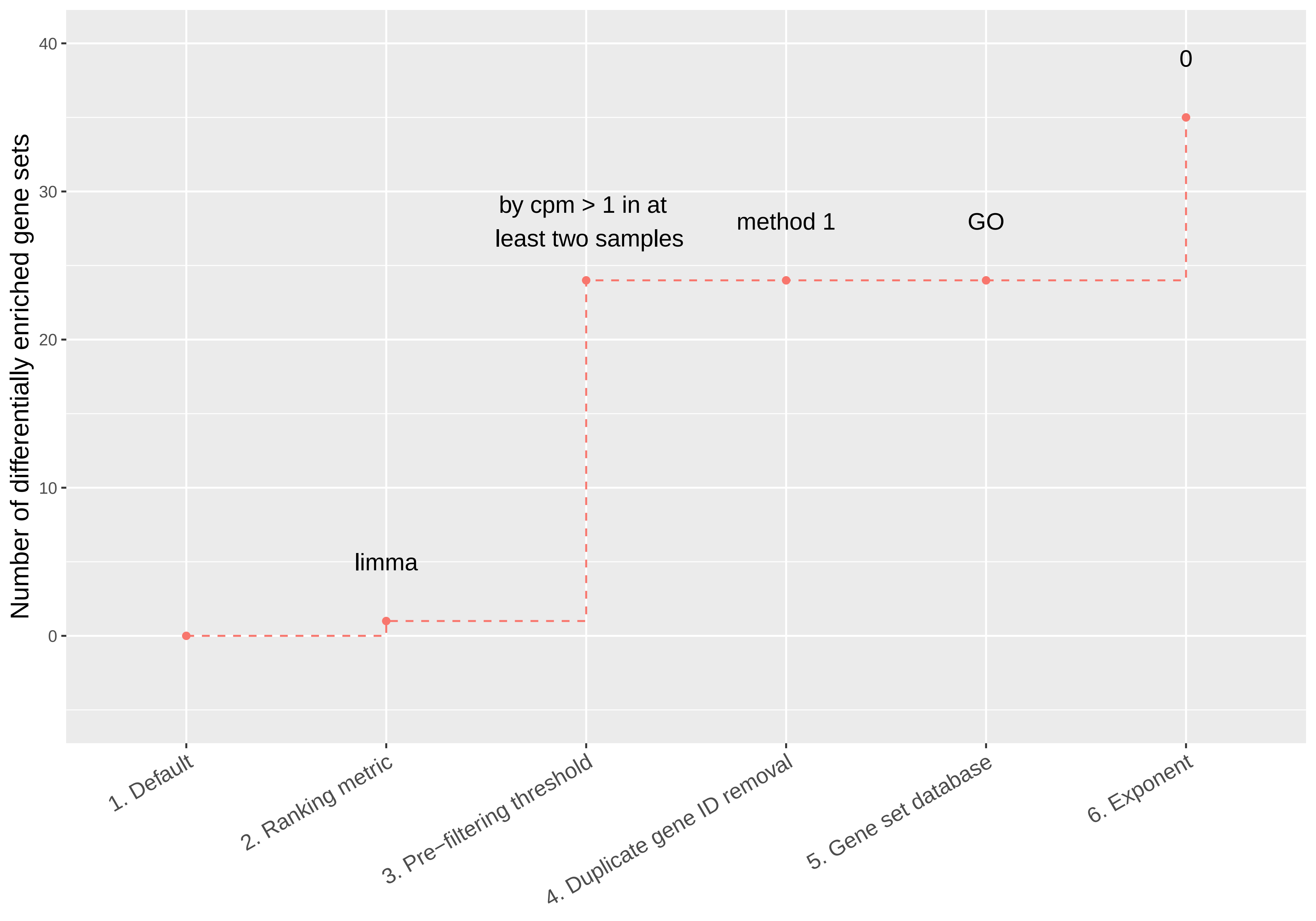

Number of differentially enriched gene sets
40
30
20
10
0
limma
by cpm > 1 in at least two samples
method 1
GO
0
1. Default
2. Ranking metric
3. Pre−filtering threshold
4. Duplicate gene ID removal
5. Gene set database
6. Exponent
