# Supplementary figures and images for: To Tweak or Not to Tweak. How Exploiting Flexibilities in Gene Set Analysis Leads to Overoptimism
Source: Biom J. 2024 Dec 19;67(1):e70016. doi: 10.1002/bimj.70016 (PMC11656295; doi:10.1002/bimj.70016)

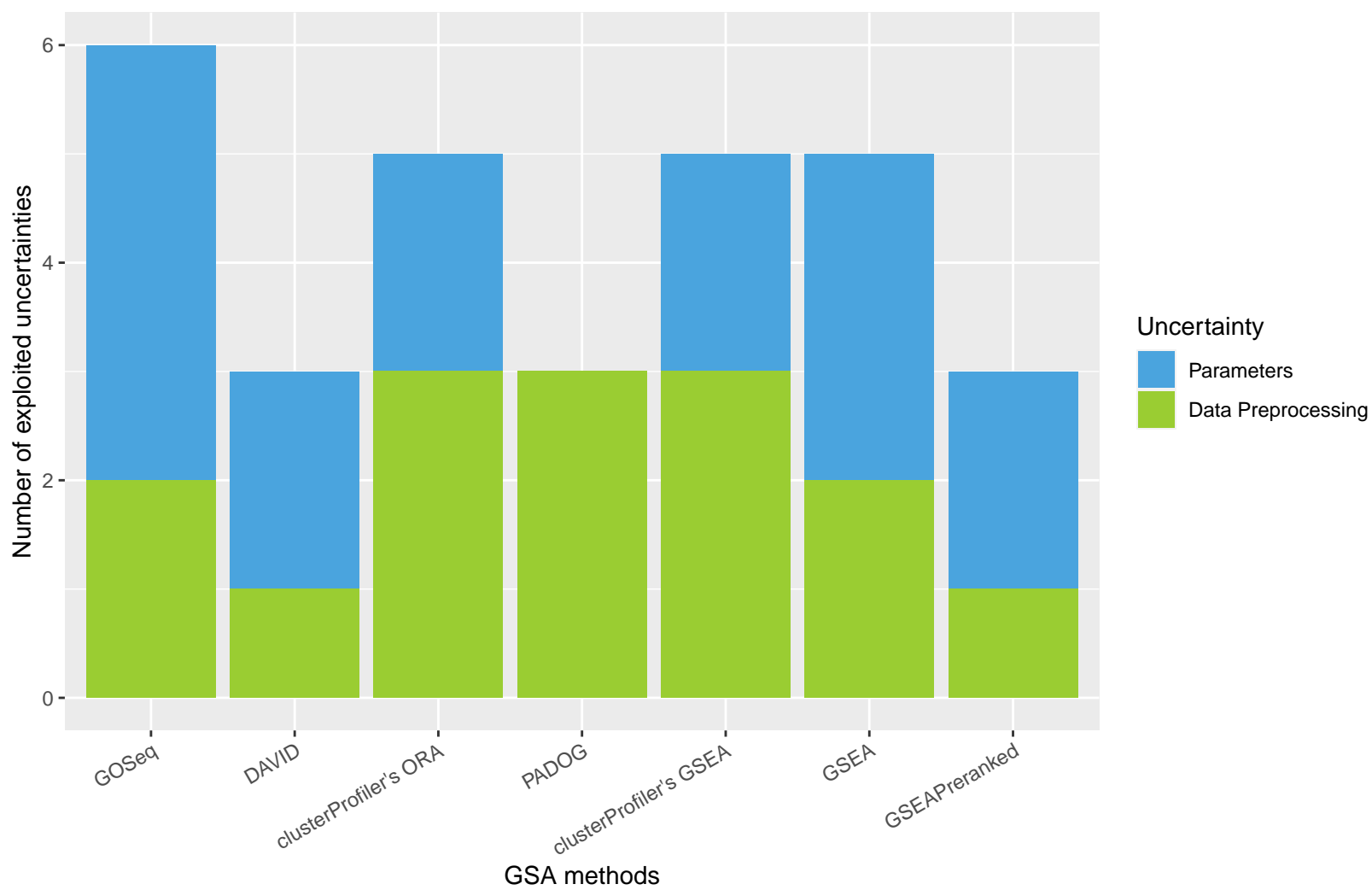

Supplement: Supplementary file 1 — Supporting Information [file BIMJ-67-e70016-s002.zip › OverOptimism_in_GeneSetAnalysis-main/Results/Figures/Figure2.pdf]

**A**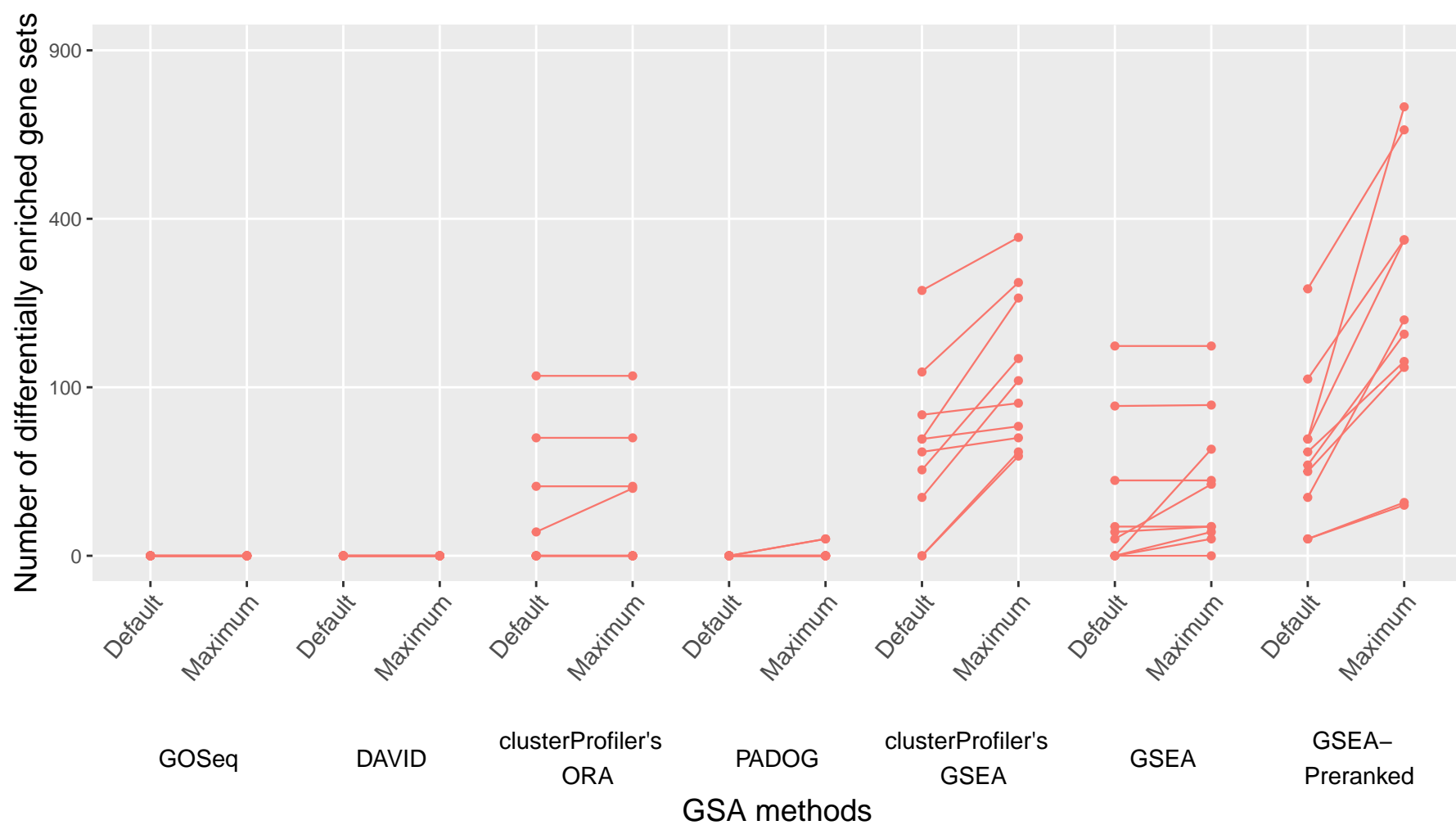**B**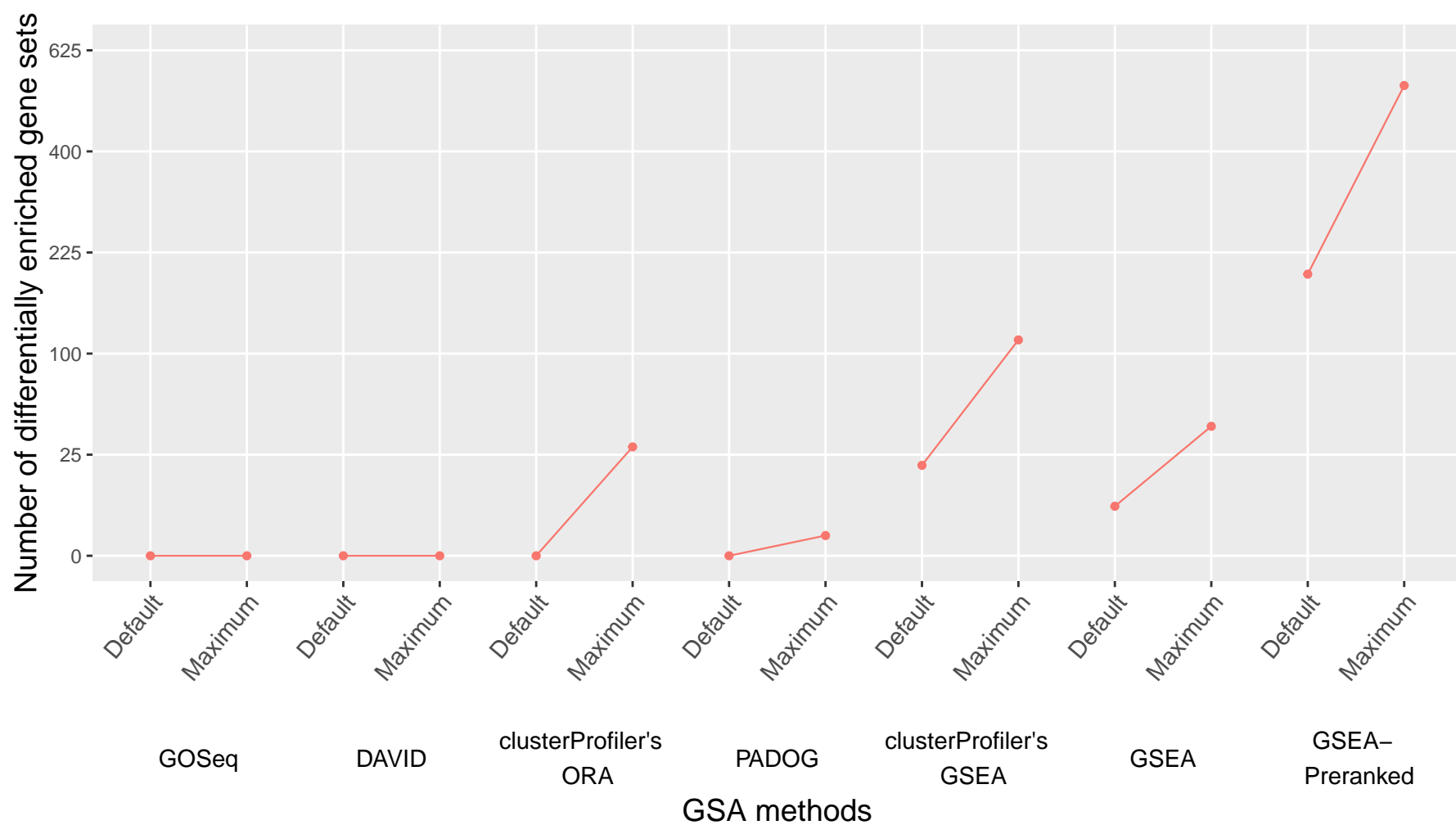

Supplement: Supplementary file 1 — Supporting Information [file BIMJ-67-e70016-s002.zip › OverOptimism_in_GeneSetAnalysis-main/Results/Figures/Figure3.pdf]

**A**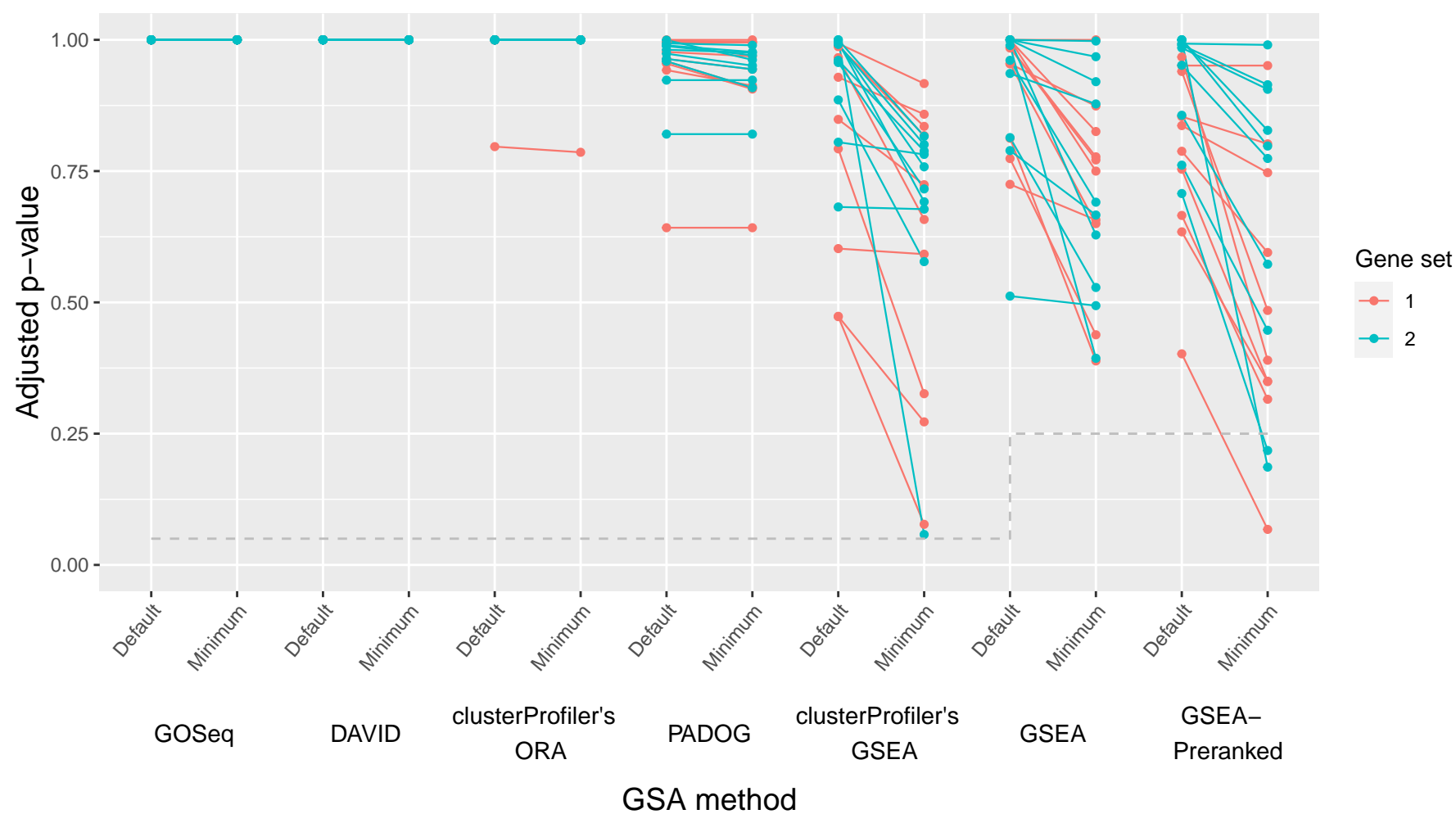**B**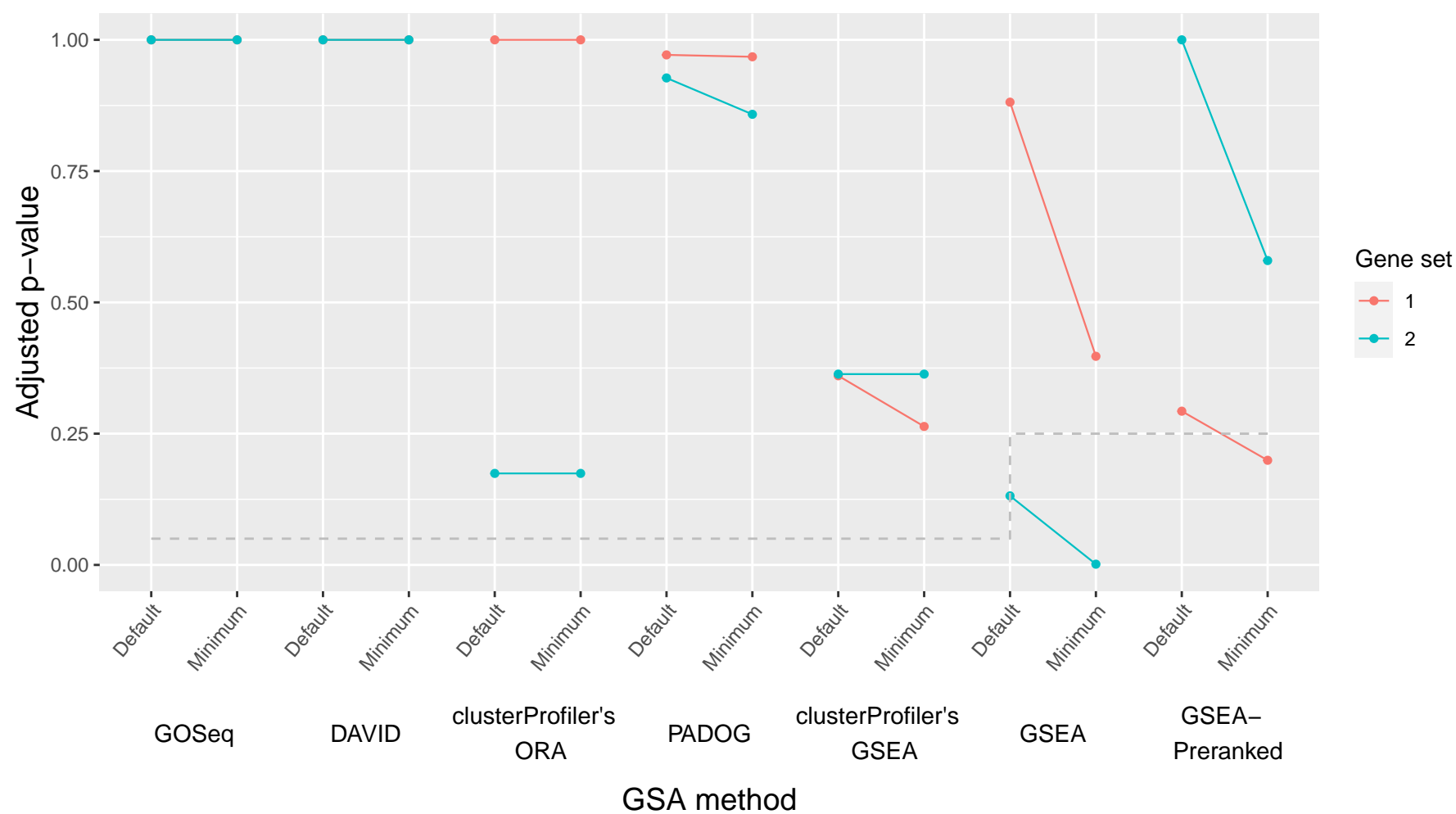

Supplement: Supplementary file 1 — Supporting Information [file BIMJ-67-e70016-s002.zip › OverOptimism_in_GeneSetAnalysis-main/Results/Figures/Figure4.pdf]

**A**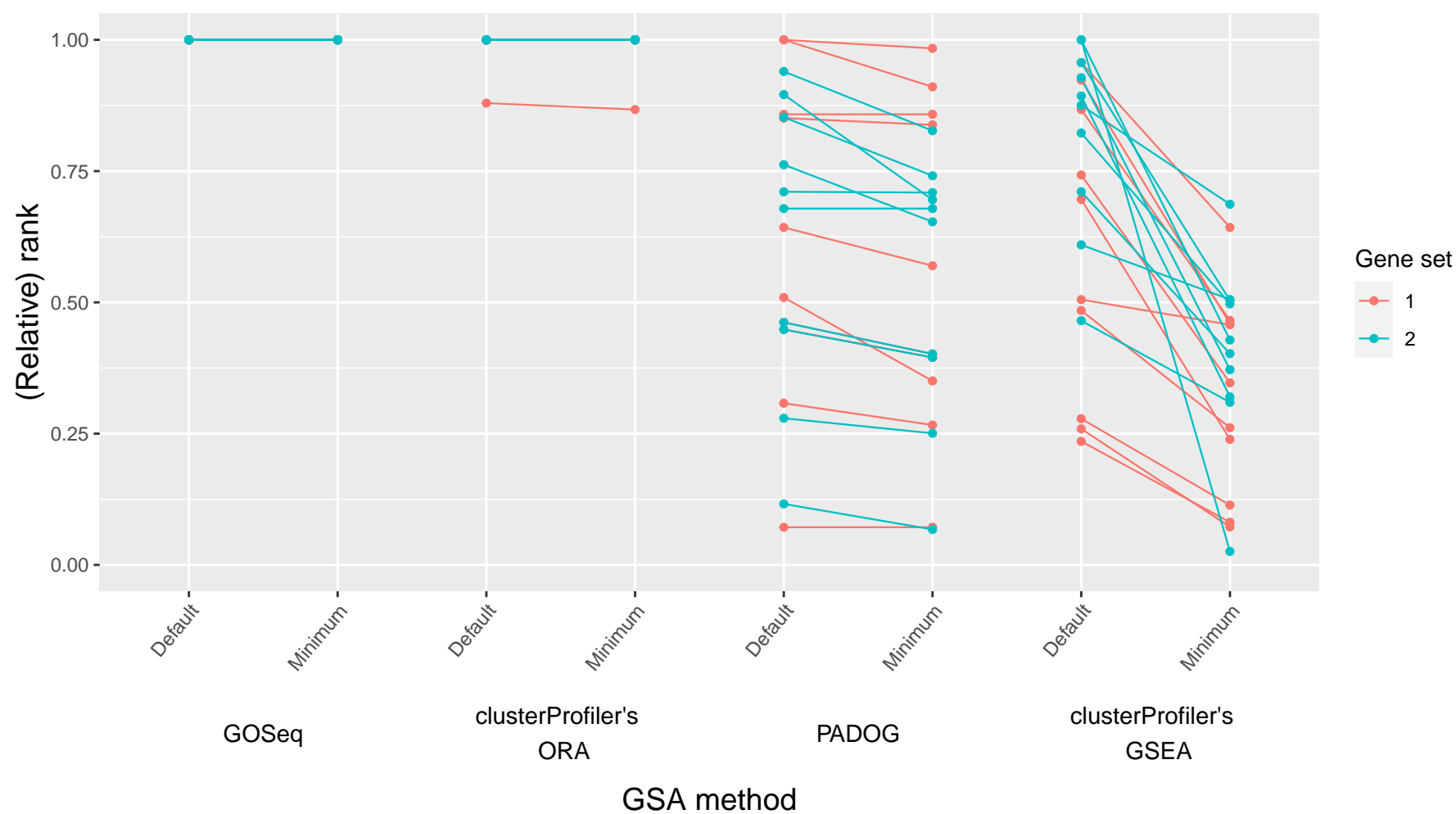**B**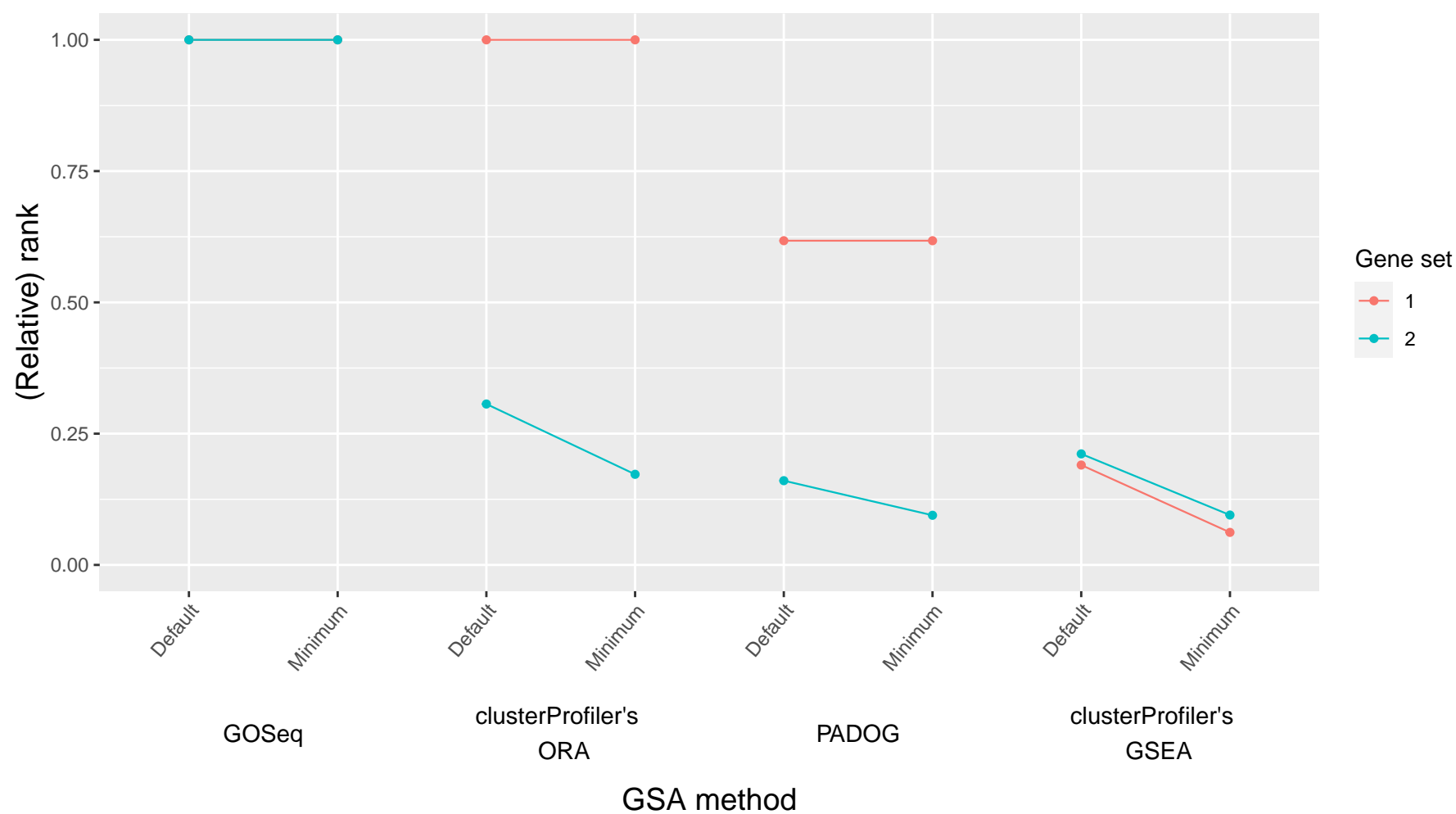

Supplement: Supplementary file 1 — Supporting Information [file BIMJ-67-e70016-s002.zip › OverOptimism_in_GeneSetAnalysis-main/Results/Figures/Figure5.pdf]

**A**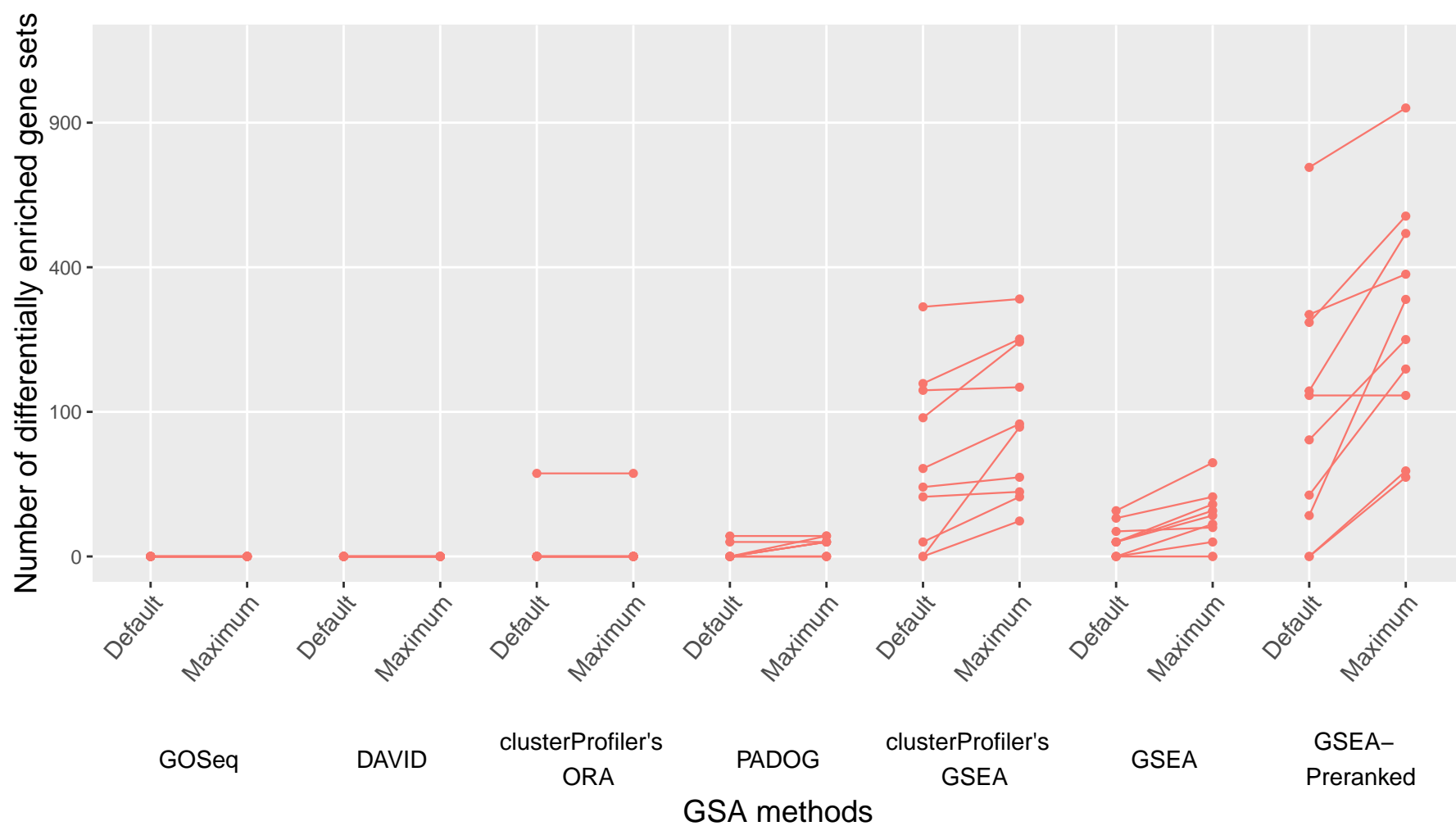**B**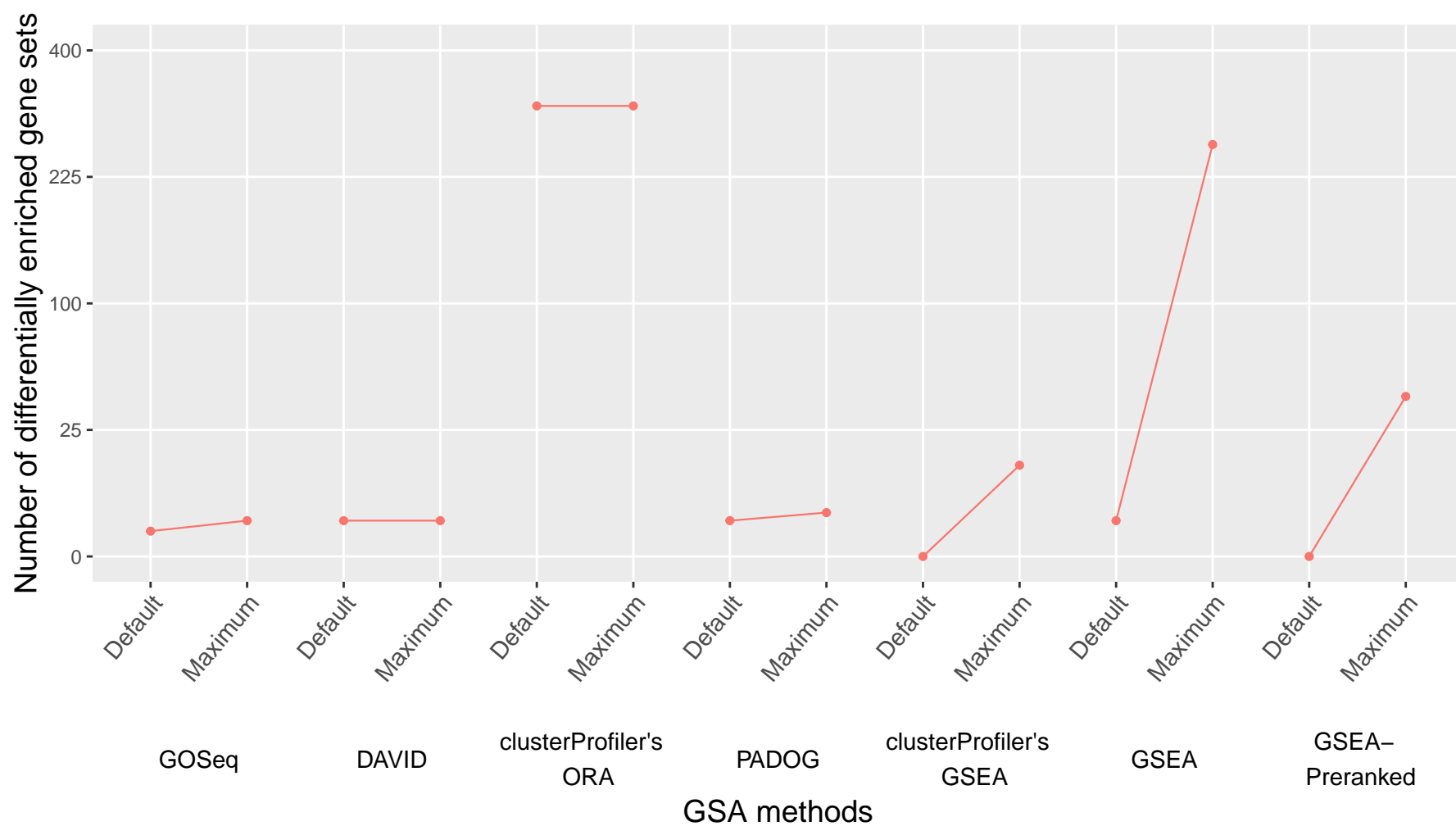

Supplement: Supplementary file 1 — Supporting Information [file BIMJ-67-e70016-s002.zip › OverOptimism_in_GeneSetAnalysis-main/Results/Figures/FigureS2.pdf]

**A**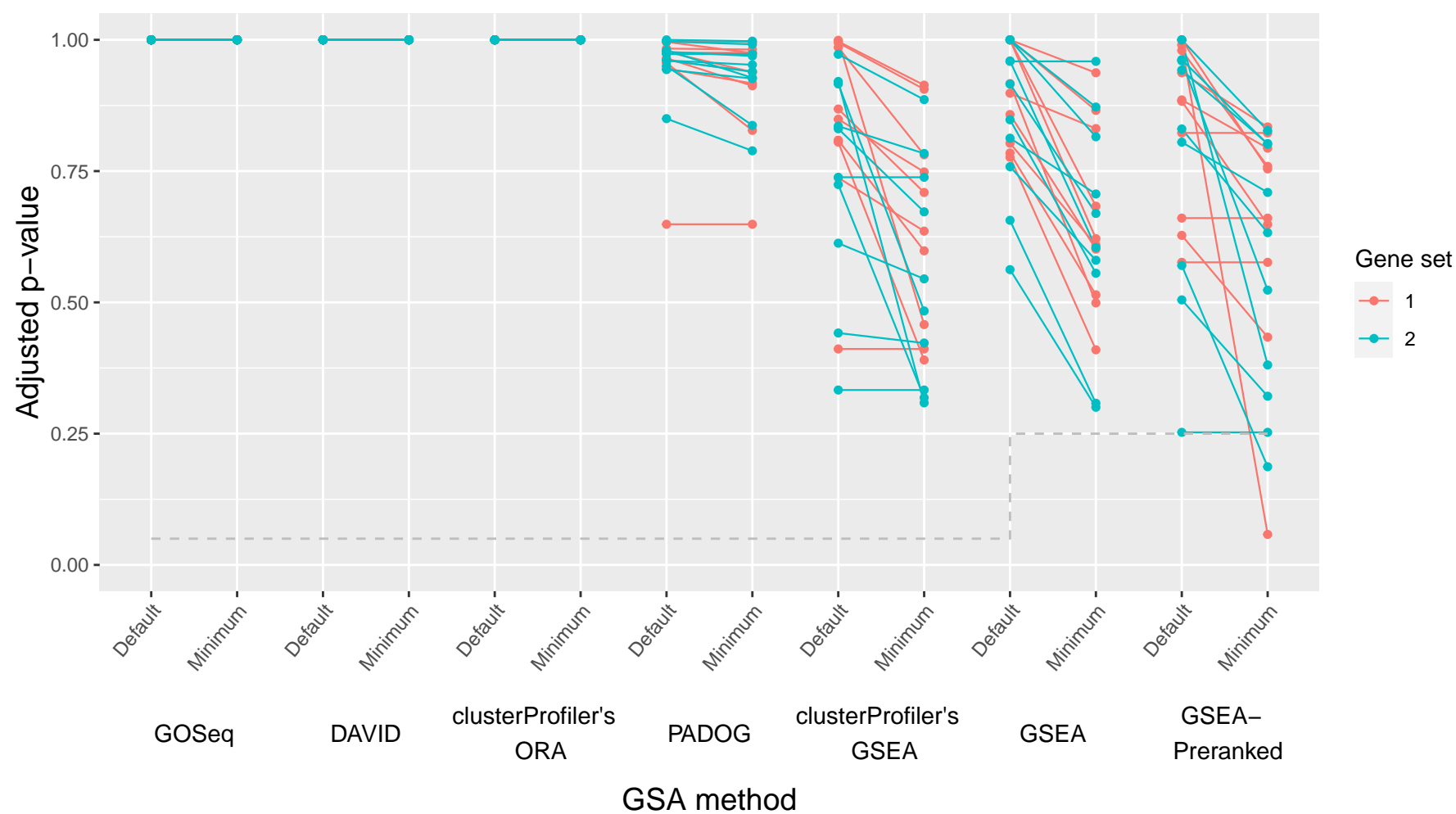**B**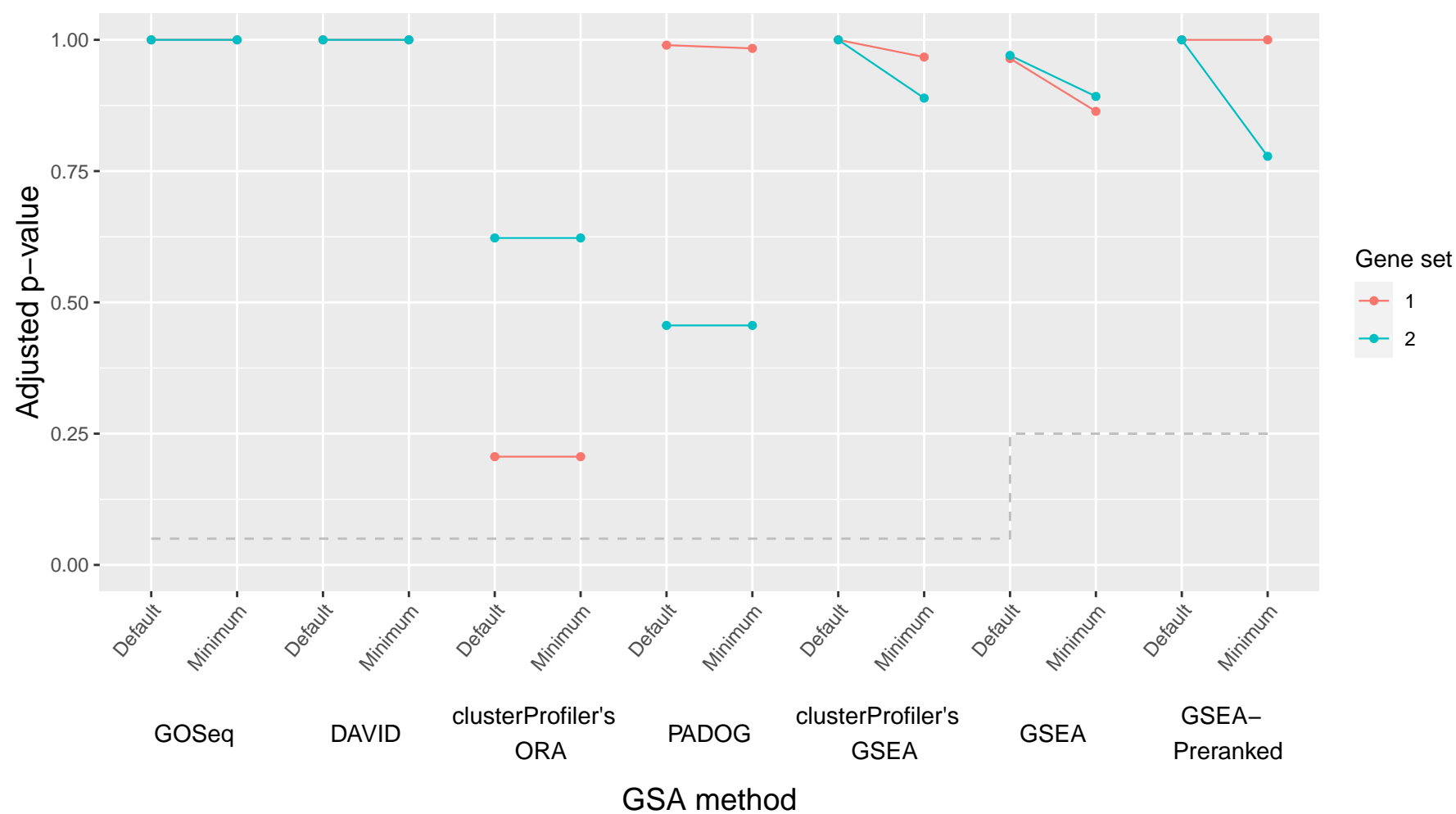

Supplement: Supplementary file 1 — Supporting Information [file BIMJ-67-e70016-s002.zip › OverOptimism_in_GeneSetAnalysis-main/Results/Figures/FigureS3.pdf]

**A**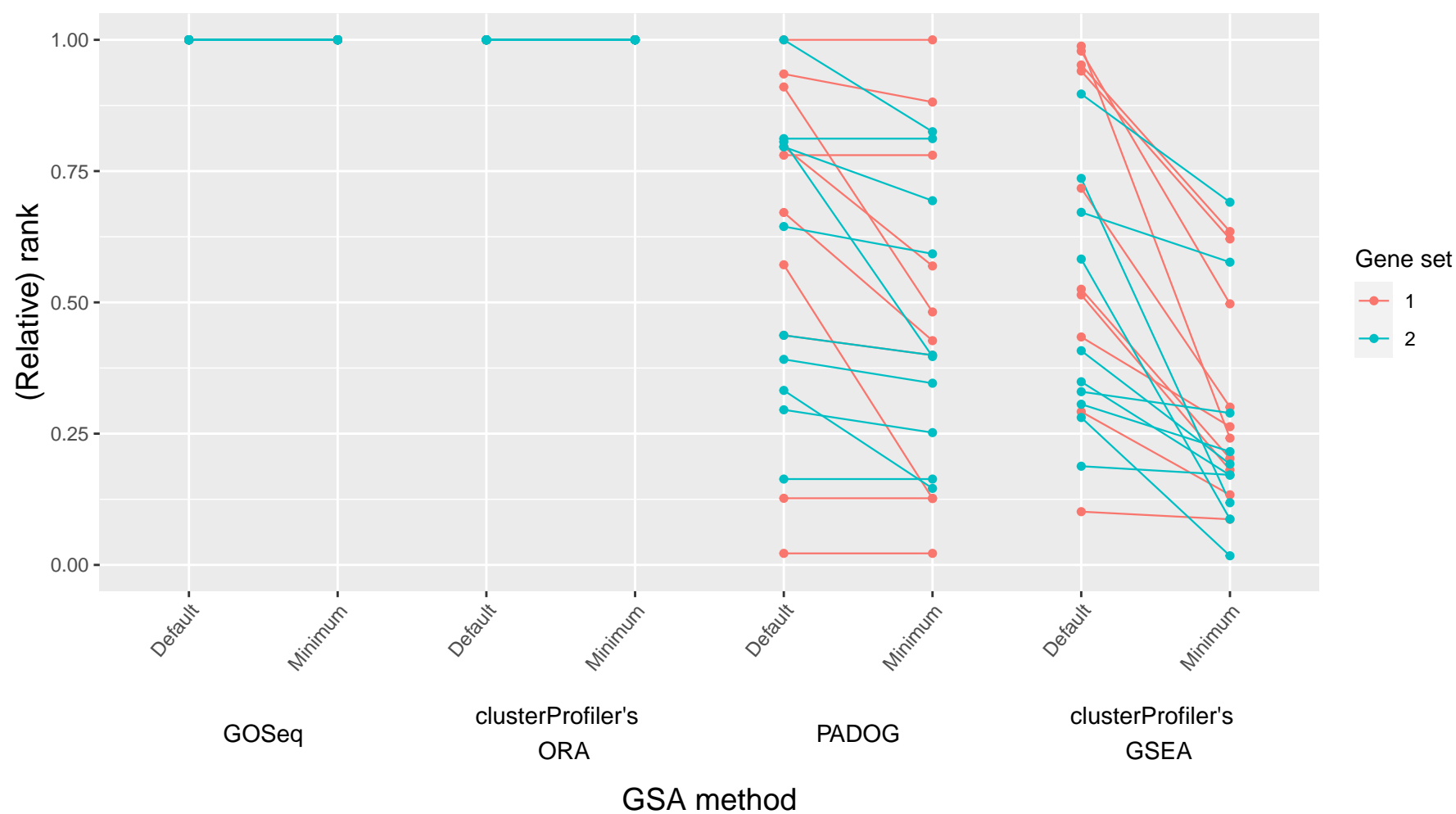**B**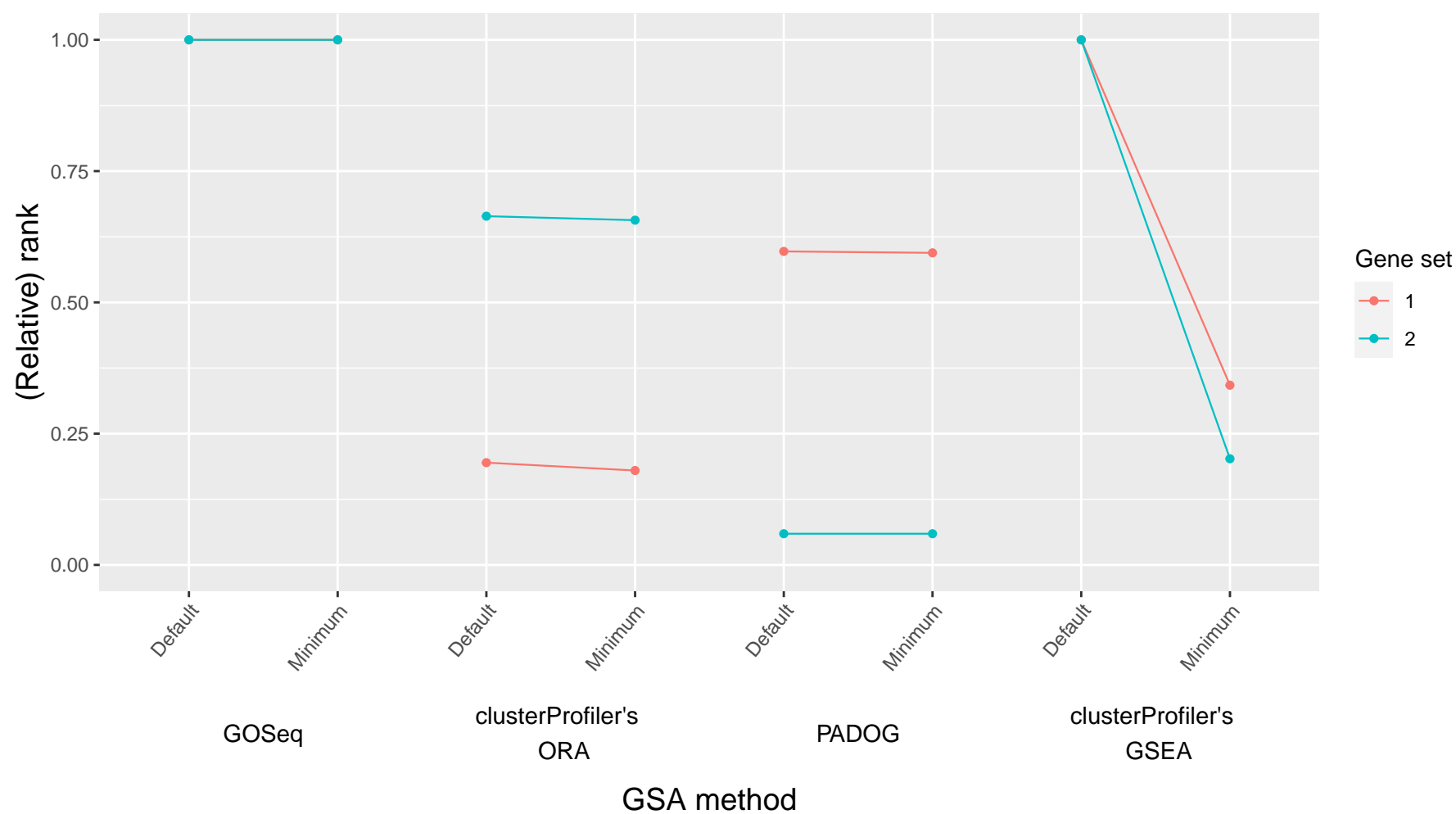

Supplement: Supplementary file 1 — Supporting Information [file BIMJ-67-e70016-s002.zip › OverOptimism_in_GeneSetAnalysis-main/Results/Figures/FigureS4.pdf]
