# Supplementary material for: To Tweak or Not to Tweak. How Exploiting Flexibilities in Gene Set Analysis Leads to Overoptimism
Source: Biom J. 2024 Dec 19;67(1):e70016. doi: 10.1002/bimj.70016 (PMC11656295; doi:10.1002/bimj.70016)
Supplement: Supplementary file 2 — Supporting Information [file BIMJ-67-e70016-s001.pdf]

To tweak or not to tweak. How exploiting flexibilities in gene set analysis leads to over-optimism.

Supplementary Material

Milena Wünsch[1,2] Christina Sauer[1,2] Moritz Herrmann[1,2]
Ludwig Christian Hinske[3] Anne-Laure Boulesteix[1,2]

[1]Institute for Medical Information Processing, Biometry, and Epidemiology, Faculty of Medicine, LMU Munich
[2]Munich Center for Machine Learning (MCML)
[3]Institute for Digital Medicine, University Hospital of Augsburg December 20, 2023

Contents

1 Additional sources of uncertainty **1**

2 Exploited uncertainty **1**
2.1 Exploited data preprocessing uncertainty 2
2.2 Exploited parameter uncertainty 3

3 Design of the study **5**
3.1 Settings 5

4 Results **7**
4.1 Illustration of one optimisation process 7
4.2 Results for the Bottomly data set 8
4.2.1 Results for goal 1: maximise number of differentially enriched gene sets (DEGS) 8
4.2.2 Results for goal 2: minimise adjusted p-value of a specific gene set 10
4.2.3 Results for goal 3: minimise rank of a specific gene set 10

1 Additional sources of uncertainty

As mentioned in the main document, there are two additional sources of uncertainty in the framework of Hoffmann et al. (2021) a researcher faces when carrying out GSA. However, they occur in the process of *generating* the gene expression data (which we assume to be complete so that we do not include these uncertainties in our study).
Measurement uncertainty, on the one hand, refers to the circumstance that the measurements contained by the data set are affected by a certain amount of imprecision. *Sampling uncertainty*, on the other hand, emerges since the data set is assumed to be a random sample of the population of interest, inducing a certain variability.

2 Exploited uncertainty

In the following, we elaborate on the analytical choices affected by uncertainty in data preprocessing (to format the available gene expression data set as required by the individual GSA methods) and the parameters that we exploit in our study. The options specified as the default to the corresponding step in our study are underlined. For a more detailed description of all of these aspects, see our earlier work (Wünsch et al., 2023).

2.1 Exploited data preprocessing uncertainty

For each of the uncertain choices in data preprocessing, the set of options was acquired through an extensive review of commonly used methods and approaches in published scientific literature. If for some steps, corresponding literature is scarce or even non-existent, we extended our review to online communities. An overview of the exploited data preprocessing uncertainties per GSA method is provided in Table S1.

Pre-filtering

Pre-filtering refers to the exclusion of lowly expressed genes, i.e. genes with a low magnitude of count data. One of several reasons to pre-filter a gene expression data set is that lowly expressed genes are unlikely to be detected as differentially expressed. Pre-filtering approaches are particularly suggested alongside DE methods and often differ between them. For DESeq2, on the one hand, a manual approach is proposed in which all genes with less than a pre-specified number of read counts are excluded from further analysis. For limma (and edgeR), pre-filtering is typically performed using the function `filterByExpr()`, which excludes all genes from further analysis with less than a pre-specified counts-per-million in a pre-specified number of samples. For those GSA methods that are not preceded by a DE method, i.e. the web-based application `GSEA` as well as `PADOG`, the two just-described approaches are selected as the two options:

(i) option 1: remove all genes with less than ten read counts across all samples,
(ii) option 2: pre-filtering using edgeR's function `filterByExpr()`.

For the remaining GSA methods in our study, the options for pre-filtering, therefore, depend on the optimal DE method in the specific optimisation process, such that the options for pre-filtering in the case of DESeq2 being the optimal DE method are

(i) option 1: remove all genes with less than ten read counts across all samples,
(ii) option 2: remove all genes with less than 50 read counts across all samples.

If, on the other hand, limma is the optimal DE method for an optimisation process, then the options to pre-filtering are

(i) option 1: pre-filtering using edgeR's function `filterByExpr()`,
(ii) option 2: remove all genes which do not have at least 1 count per million in at least two samples.

Note that option 2 stems from an older user guide provided by edgeR in 2018.

Removal of duplicated gene IDs

There are a multitude of formats to identify the genes in the gene expression data set and if the format in the given data set differs from the format(s) accepted by the chosen GSA method, a conversion to the required format is necessary. This conversion results in duplications of some gene IDs which must be removed manually by the user. The two utilised options for removing these duplicates are based on our earlier work (Wünsch et al., 2023) and correspond to

(i) option 1: keep the gene ID occurring first,
(ii) option 2: in each sample, keep the rounded mean expression value of all genes associated with the duplicated gene ID.

Differential expression analysis

Differential expression analysis is typically performed for ORA methods to obtain the required input in the form of a list of differentially expressed genes. However, it is also commonly used for FCS methods which require as input a ranking of all genes from the experiment based on their magnitudes of differential expression. In our study, we utilise two popular (and parametric) DE methods, namely

(i) option 1: DESeq2 (Love et al., 2014),
(ii) option 2: voom/limma (Law et al., 2014).

As described above, we specify the options to pre-filtering in a specific optimisation process depending on the optimal DE method.

Table S1: Overview of the exploited data preprocessing steps per computational GSA method. Note that we use the term 'cP' as an abbreviation for the R package 'clusterProfiler'. As described in the main document, we have reduced the number of exploited preprocessing steps for the web-based applications DAVID, GSEA, and GSEAPreranked since the corresponding optimisation processes are performed by hand.

	ORA methods			FCS methods			
Preprocessing step	DAVID	GOSeq	cP's ORA	GSEA	GSEAPreranked	PADOG	cP's GSEA
Pre-filtering		×	×	×		×	×
Removal of duplicated gene IDs			×			×	×
Differential expression analysis	×	×	×		×		×
Transformation (and normalisation)				×		×	

Transformation (and normalisation)
Those FCS methods investigated in our study that require as input the gene expression data set as a whole were initially developed for microarray measurements. Therefore, they assume different characteristics than those that are rooted in RNA-Seq measurements. A common way to handle this discrepancy is to transform the RNA-Seq data such that the characteristics of the transformed data (approximately) match those of microarray measurements. We have selected two methods for RNA-Seq transformation based on our earlier work (Wünsch et al., 2023), namely

(i) option 1: voom transformation as part of the work of Law et al. (2014),
(ii) option 2: variance stabilising transformation as proposed by Love et al. (2014).

Note that both transformation methods additionally normalise the RNA-Seq measurements. For a more detailed description of both transformation methods, see our earlier work (Wünsch et al., 2023).

2.2 Exploited parameter uncertainty

In the following, we elaborate on the five parameter choices affected by uncertainty that we exploit in our study. For an additional overview, see Table S2. While some parameters are flexible across ORA and FCS, others are specific to one of the two general approaches. In particular, no parameter can be chosen flexibly for all considered GSA methods. For all of these parameters apart from the gene set database, a default value is proposed by the corresponding method. A detailed description of these parameters can be found in our earlier work (Wünsch et al., 2023). Note that for each uncertain parameter, we choose the number of options within a range that we regard as realistic considering that a (well-intentioned) researcher is unlikely to try indefinitely many options.

Geneset database
In this work, we focus on the choice between the following two commonly used gene set databases which are offered by (almost) all of the considered GSA methods:

(i) option 1: Gene Ontology ('GO') with subontology 'Molecular Function' (Ashburner et al., 2000; Aleksander et al., 2023),
(ii) option 2: KEGG (Kanehisa and Goto, 2000; Kanehisa et al., 2023).

We do not make use of the opportunity to upload a user-defined gene set database which is offered by some GSA methods. Note that the individual GSA methods do not automatically refer to identical versions of the respective gene set database since the methods are not maintained in identical time intervals. As a consequence, the number of gene

sets (and therefore also the set of gene sets) contained by a specific gene set database can differ between the considered GSA methods.

Universe

The flexible choice of the universe (also called 'background') is specific to the GSA methods classified as ORA since typically only a subset of the genes from the experiment are provided as input for the corresponding methods. This leads to a loss of information on the entirety (i.e. 'universe') of the genes. However, in ORA, this information is typically required as the 'population' in the hypergeometric distribution which serves as the underlying null distribution. The information on the universe must therefore be obtained alternatively. In our study, we utilise the following options as the universe:

(i) option 1: all genes that are part of the chosen gene set database,
(ii) option 2: all genes from the initial gene expression data that are tested for differential expression.

`GOSeq` constitutes an exception to the 'regular' ORA methods as its required input corresponds to all genes from the experiment labelled by their status of differential expression. This means that the information on the entirety of the genes is, in contrast to the 'regular' ORA methods, available. The options of the universe utilised in our study for `GOSeq` are therefore the following:

(i) option 1: all genes from the input that are members to at least one gene set from the chosen gene set database,
(ii) option 2: all genes from the input.

Method

`GOSeq` is the only method among the selection in which the user can modify the method to obtain the p-values of enrichment of the gene sets. In our study, we exploit the following options for `GOSeq`:

(i) option 1: Wallenius distribution (Wallenius, 1963),
(ii) option 2: resampling method.

The Wallenius distribution is an approximative approach and an extension to the hypergeometric distribution while the alternative resampling method computes the p-values of enrichment in a manner similar to FCS methods.

Gene-level statistic

For FCS methods, the gene-level statistic is the metric (i.e. 'formula') used to generate the ranking of the genes based on each of their magnitude of differential expression between the conditions. For those FCS methods that generate the ranking internally (`GSEA` and `PADOG` in our study), it is generated directly from the initial gene expression data set. For the remaining FCS methods which require the user to create the ranking externally as part of data preprocessing (`GSEAPreranked` and `clusterProfiler`'s GSEA), it is typically created from the results of differential expression analysis. For the former, the choice of a gene-level statistic often presents a flexible parameter within the method, contributing to parameter uncertainty. The uncertain choice of a gene-level statistic for the latter, on the other hand, indicates data preprocessing uncertainty as opposed to parameter uncertainty (note however, in our study, we use a fixed gene-level statistic for these FCS methods in the data preprocessing).

Note that for `PADOG`, the gene-level statistic does not present any flexibility. Therefore, the web-based application `GSEA` is the only FCS method offering flexibility in the parameter 'gene-level statistic'. The corresponding options are

(i) option 1: signal-to-noise ratio,
(ii) option 2: t-statistic,
(iii) option 3: difference of classes.

Weight

The parameter 'weight' is related to the strength of the contribution of each gene in the assessment of differential enrichment of a given gene set. For `GOSeq`, the weight of each gene enters the analysis through a probability weighting function. In our studies, we utilise the following options for `GOSeq`

(i) option 1: assign a higher weight to genes with shorter transcript length,
(ii) option 2: assign a higher weight to the genes with a lower gene expression level in the RNA-Seq gene expression data set.

Table S2: Overview of the exploited parameters per computational GSA method. Note that 'cP' is the abbreviation for the R package clusterProfiler. The asterisk '*' indicates that the choice of gene set database is exploited for optimisation goal 1 only.

	ORA methods			FCS methods			
Preprocessing step	DAVID	GOSeq	cP's ORA	GSEA	GSEAPreranked	PADOG	cP's GSEA
Gene set database*	×	×	×	×	×		×
Universe	×	×	×				
Method		×					
Gene-level statistic				×			
Weight		×		×	×		×

Option 1 accounts for the circumstance that genes with longer transcript length are more likely to be detected as differentially expressed, leading to a higher likelihood of detection of differential enrichment of those gene sets whose members are generally longer in transcript length. On the other hand, option 2 accounts for all possible biases leading to a decreased statistical power in detecting the gene sets as differentially enriched that contain many genes with a low overall expression level.

In the context of the FCS methods, the weight of the genes can be modified for those methods that are based on the method Gene Set Enrichment Analysis (GSEA, GSEAPreranked, and clusterProfiler's GSEA), namely by adapting the exponent in the calculation of the enrichment score (see Subramanian et al. (2005) or Wünsch et al. (2023) for a more detailed description). In our study, we utilise as options for the exponent value

(i) option 1: exponent 1. Weight each gene by its absolute value of the gene-level statistic.
(ii) option 2: exponent 0. Assign each gene the same weight.
(iii) option 3: exponent 1.5. Weight each gene by the 1.5-times exponentiated value of the gene-level statistic.
(iv) option 4: exponent 2. Weight each gene by the squared value of the gene-level statistic.

3 Design of the study

3.1 Settings

Gene expression data sets
For our study, we have selected two RNA-Seq data sets based on the following criteria:

(i) the conditions of the samples are known and binary,
(ii) there are at least ten samples per condition,
(iii) the sample sizes per condition are similar,
(iv) the gene expression measurements stem from an organism supported by each of the investigated computational methods.

Criteria (ii) and (iii) ensure that we can generate a sufficient number (i.e. ten) of random permutations of the true sample labels whose assignments to the individual samples differ from the true assignments in sufficiently many cases.

Gene sets (for goals 2 and 3)
For illustration, we initially intended to select gene sets for the Pickrell and Bottomly data set, respectively, from published application papers that are similar in terms of the underlying biological context and research question. As the match for the Pickrell data set, we chose the work of Lopes-Ramos et al. (2020) who use GSEAPreranked to identify sex-biased enrichment in 29 tissue types (28 solid tissues, including adipose (subcutaneous), thyroid, and

whole blood), based on 8279 tissue samples. The data set contains gene expression measurements of $n = 188$ female and $n = 360$ male subjects and the expression profiles of 30243 genes. As the gene sets whose adjusted p-value and rank we attempt to optimise for goals 2 and 3, we chose two gene sets with the highest magnitude of differential enrichment between males and females in the tissue 'whole blood'. This selection resulted in the gene sets *Demethylation* and *T cell mediated immunity*, both from gene set database GO with subontology 'Biological Process'.
For the RNA-Seq data set provided by Bottomly et al. (2011), we selected the work of Kraus et al. (2012) as the corresponding match. Their work includes three Panther gene ontology (GO) analyses that compare the gene expression level between entire mouse embryos of four strains, eviscerated mouse embryos of four strains, and eviscerated embryos of eleven strains. In their work, gene expression is measured in the form of microarray measurements. Note that mouse strain 'C57BL/6J' is among the considered mouse strains in all three analyses, whereas strain 'DBA/2J' is not included. We have selected the two gene sets with the highest relevance in all three results, namely *Metabolic process* and *Cellular Process*. Both gene sets are provided by the gene set database Gene Ontology with subontology Biological Process.
However, several issues in the attempt to minimise the adjusted p-values and ranks of these gene sets selected for the Pickrell and Bottomly data set force us to resort to alternative gene sets in these cases. For an overview of the selected gene sets for each GSA method and gene expression data set, see Table S3.
Firstly, when providing a gene expression data set stemming from the mouse organism, `GSEA` and `GSEAPreranked` internally convert the mouse gene IDs to orthologous human gene IDs. Differential enrichment is, therefore, assessed for 'human' gene sets. Consequently, we cannot work with either of the 'mouse' gene sets extracted from Kraus et al. (2012) when working with the Bottomly data set. In these cases, we, therefore, use the corresponding 'human' gene sets from Lopes-Ramos et al. (2020), instead.
For `clusterProfiler`'s GSEA, neither of the two gene sets from Kraus et al. (2012) are included by GO, forcing us to proceed in the same manner as with `GSEA` and `GSEAPreranked` for the Bottomly data set. For `clusterProfiler`'s ORA, both gene sets from Kraus et al. (2012) are also not included in any of the results tables. However, we cannot assess whether both gene sets are indeed not even provided by the gene set database GO since the results table provided by `clusterProfiler`'s ORA generally only include those gene sets containing at least one differentially expressed gene set from the input. However, because both gene sets are not included by GO when working with `clusterProfiler`'s GSEA, we also resort to both gene sets matching the Pickrell data set for `clusterProfiler`'s ORA. Finally, `PADOG` does not offer the gene set database GO internally so we have to resort to KEGG, leading to the choice of the gene sets 'Primary immunodeficiency' and 'Graft versus host disease'. Both gene sets are among the most differentially enriched KEGG pathways in the tissue 'whole blood'. Since when using `PADOG`, the considered gene sets are the same for gene expression data stemming from human and mouse, we use these gene sets for both gene expression data sets.

Table S3: The gene sets whose adjusted p-values and ranks are optimised for goals 2 and 3, respectively. The representation is clustered by the GSA methods for which the same gene sets are optimised.

GSA method	Gene sets (1 and 2) optimised in Pickrell data set	Gene sets (1 and 2) optimised in Bottomly data set
`GOSeq`	T cell mediated immunity<br>Demethylation	Cellular process<br>Metabolic process
`DAVID`	T cell mediated immunity<br>Demethylation	Cellular process<br>Metabolic process
`clusterProfiler`'s ORA	T cell mediated immunity<br>Demethylation	T cell mediated immunity<br>Demethylation
`PADOG`	Primary immunodeficiency<br>Graft versus host disease	Primary immunodeficiency<br>Graft versus host disease
`clusterProfiler`'s GSEA	T cell mediated immunity<br>Demethylation	T cell mediated immunity<br>Demethylation
`GSEA`	T cell mediated immunity<br>Demethylation	T cell mediated immunity<br>Demethylation
`GSEAPreranked`	T cell mediated immunity<br>Demethylation	T cell mediated immunity<br>Demethylation

4 Results

4.1 Illustration of one optimisation process

We describe the progression of one specific optimisation process resulting from the setting consisting of

(i) goal 1: maximise the number of differentially enriched gene sets
(ii) gene expression data set: Pickrell data set
(iii) sample labels: random sample label permutation 6
(iv) method: `clusterProfiler`'s GSEA.

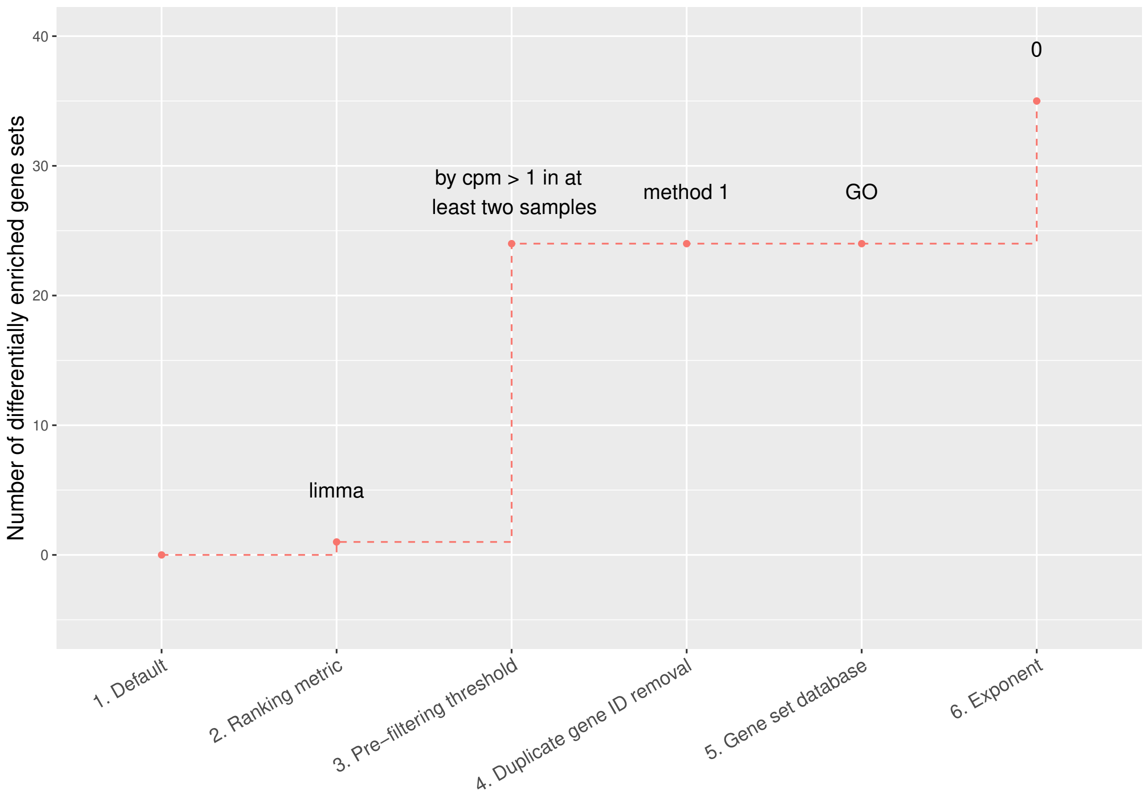

Figure S1: Illustration of the progression of one optimisation process following goal 1 in a step diagram. The individual optimisation steps are displayed on the x-axis and the corresponding optimal analytical choices are indicated above the steps. This optimisation process takes place in the setting of optimisation goal 1, the Pickrell data set with the sample labels corresponding to permutation 6, and `clusterProfiler`'s GSEA.

As displayed in Figure 2 in the main document, we exploit three analytical choices associated with data preprocessing uncertainty and two associated with parameter uncertainty. The first three choices consist of the DE method to generate the input ranking of the genes, the approach to pre-filtering, and the approach to removing the duplications of the gene IDs (resulting from gene ID conversion). On the other hand, we exploit the choice of the gene set database and the exponent in the computation of the enrichment score as part of parameter uncertainty. The corresponding default choices are

(i) DE method: DESeq2,
(ii) pre-filtering approach: remove all genes with less than 50 read counts across all samples,
(iii) approach to the removal of duplicated gene IDs: keep the corresponding gene ID among the duplicates that occurs first,
(iv) gene set database: GO (with subontology Molecular Function),

(v) exponent value: 1, i.e. each gene is weighted by their absolute value of the gene-level statistic in the computation of the enrichment score.

From Figure S1, in which a graphical illustration of the progression of the optimisation process is provided, we observe that 0 differentially enriched gene sets result from these default choices.

Step 1: optimise the choice of the DE method

When specifying the alternative method limma as the DE method from which the ranking of the genes is generated, the number of DEGS increases to 1 compared to when DESeq2 is specified as the DE method. We therefore set **limma** as the optimal choice of the DE method and update the current optimal results to 1 **DEGS**.

Step 2: optimise the choice of the pre-filtering approach

As described in Section 2.1, the options to pre-filtering depend on the optimal DE method as determined in the previous step of the optimisation process. The number of DEGS of 1 at the beginning of this second optimisation step is therefore based on the default pre-filtering approach for limma. This approach corresponds to pre-filtering using the function *filterByExpr()* from the `R` package `edgeR` (Robinson et al., 2010) while the alternative option consists of removing all genes which do not exceed 1 'count-per-million' in at least two samples. This alternative pre-filtering approach results in 24 DEGS, therefore exceeding 1 DEGS from the default pre-filtering approach. We, therefore, set **pre-filtering by cpm** > 1 **in at least two samples** as the optimal pre-filtering approach and update the current optimal results to 24 **DEGS**.

Step 3: optimise the choice of the approach to the removal of duplicated gene IDs

The alternative approach to the removal of duplicated gene IDs, in which for each duplication the (rounded) mean of read counts of associated genes is kept, cannot exceed the default approach in terms of the number of DEGS. The optimal approach to pre-filtering in this optimisation process therefore corresponds to the default approach of **keeping the gene ID that occurs first** and the current optimal results remain at 24 **DEGS**.

Step 4: optimise the choice of the gene set database

The alternative gene set database KEGG does not lead to an increased number of DEGS compared to the default gene set database GO (with subontology 'Molecular Function'). The current optimal results therefore remain at 24 DEGS and the optimal gene set database for this optimisation process is **GO (with subontology 'Molecular Function')**.

Step 5: optimise the choice of the exponent in the calculation of the enrichment score

Assigning each gene the same weight in the computation of the enrichment score (as opposed to weighting each gene by its absolute level of the gene-level statistic) leads to an increase in the number of DEGS to 35, which is the highest among all alternative options. Consequently, we set **exponent 0** as the optimal exponent choice and the **final optimal results** are then 35 **DEGS**.

Summary

Through the exploitation of uncertainty in five analytical choices (three choices in data preprocessing and two parameter choices), we can tweak the number of DEGS from 0 to 35.

4.2 Results for the Bottomly data set

4.2.1 Results for goal 1: maximise number of differentially enriched gene sets (DEGS)

For an overview of the results, inspect Figure S2.

Random sample label permutations: For `GOSeq` and `clusterProfiler`'s ORA, we observe results similar to the Pickrell data set, namely that no over-optimistic results can be induced in any of the permutations. In particular, the numbers of DEGS amount to 0 before and after exploiting any uncertainties in the vast majority of permutations. For `PADOG`, our observations also agree with those for the Pickrell data set. An increase from an initial number of 0 DEGS can be obtained for some of the permutations through the choice of the pre-filtering approach and the method to transform the RNA-Seq data. As with the Pickrell data set, these do not exceed 2 DEGS after exploitation of uncertainty in any of the permutations.

As with the Pickrell data set, the GSEA-based methods, again, indicate a higher potential for over-optimistic results compared to the remaining methods. Among those, it is particularly high for `GSEAPreranked`, for which notable increases are obtained in all but one sample label permutation. The same set of analytical choices triggering increases can be observed as in the Pickrell data set, namely the choice of the DE method and assigning equal weights to all genes in the computation of the enrichment score. This way, a particularly strong increase from 8 to 316 DEGS increase can be triggered in one permutation.

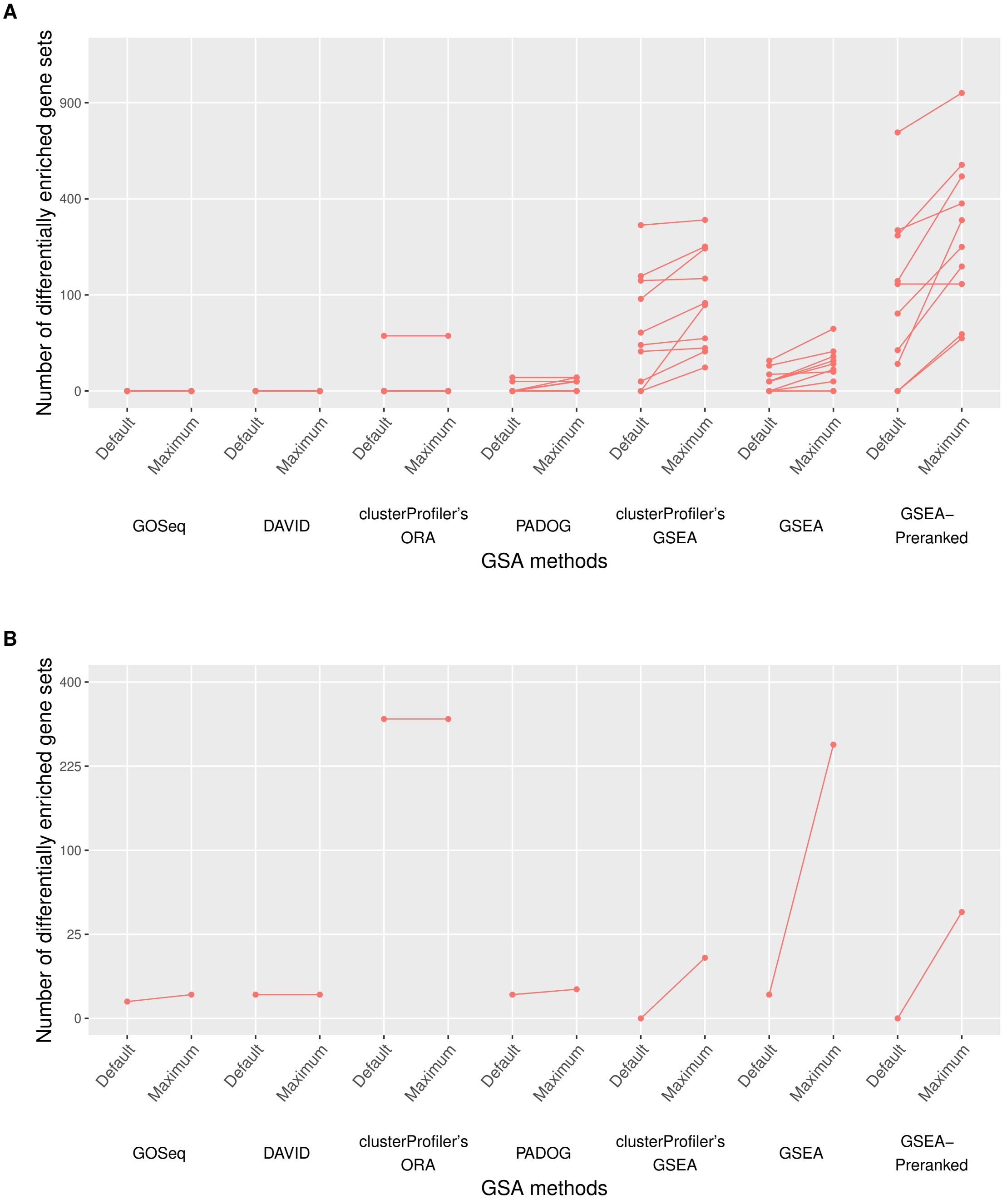

Figure S2: Goal 1: The optimised numbers of differentially enriched gene sets ('Maximum') in the Bottomly data set, obtained through the exploitation of uncertainty, are compared to the corresponding numbers resulting from the default analytical choices ('Default'). For each optimisation process, the associated optimised and the default number are connected through a line. (A) presents the results for the ten random permutations and (B) for true sample labels. On the x-axis, the individual methods investigated in the context of goal 1 are displayed. Note the transformation of the scale of the y-axis to represent the number of DEGS of different orders of magnitude.

For clusterProfiler's GSEA and the web-based method GSEA, we also observe several notable increases in the number of DEGS. While in absolute terms, the induced increases are generally stronger for the former method, several increases from an initial number of 0 (or close to 0) to considerably higher numbers can be triggered for the web-based method GSEA, which can be of considerable value to a researcher.

True sample labels: The number of DEGS can be tweaked for all considered methods apart from DAVID and clusterProfiler's ORA. For the latter, however, the number of DEGS, exceeding 300, is already substantial. For GOSeq and PADOG, an increase of 1 DEGS is induced through the choice of the method to transform the RNA-Seq data and the choice of the gene set database KEGG, respectively. Given the initial number of 2 and 1 DEGS, respectively, this increase is high in relative terms. Considerable 'absolute' increases can be achieved for the three GSEA-based methods. For GSEAPreranked and clusterProfiler's GSEA, respectively, a notable increase from 0 is obtained solely through weighting each gene equally in the computation of the enrichment score. The highest increase among all methods is obtained for the web-based application GSEA through the exploitation of uncertainty in several analytical choices in data preprocessing and the parameters. Thereby, the strongest increase is induced through the modification of the weighting pattern of the genes in the computation of the enrichment score.

4.2.2 Results for goal 2: minimise adjusted p-value of a specific gene set

Random sample label permutations: For GOSeq, DAVID, and clusterProfiler's GSEA, we are not able to induce any over-optimistic results through the exploitation of data preprocessing and parameter uncertainty in the context of goal 2. In particular, for these three methods, the adjusted p-values of both gene sets amount to 1 before and after the exploitation of uncertainty in all of the permutations. For PADOG, the adjusted p-values resulting from the default choices are close to 1 in the vast majority of permutations. While they can be tweaked for several permutations, particularly through the choice of the pre-filtering method and the method to transform the RNA-Seq data, the effect is mostly negligible, resulting in tweaked adjusted p-values that are still close to 1.

For clusterProfiler's GSEA, web-based GSEA and GSEAPreranked, we observe moderate to notable decreases in the adjusted p-values (/q-values) for both gene sets and in the majority of permutations. While for the former two methods, neither of them transforms an initially non-significant adjusted p-value (/q-value) into a significant one, a significant q-value of enrichment is obtained in one permutation for gene set 1 and gene set 2, respectively, for GSEAPreranked. For the latter gene set particularly, the choice of the DE method as part of data preprocessing and the modification of the weighting pattern of the genes in the computation of the enrichment score tweak the q-value from 0.99 to 0.06.

True sample labels: For all of the considered methods and the respective two gene sets, we observe negligible to at most moderate decreases in the respective adjusted p-values (/q-values), such that the tweaked adjusted p-values (/q-values) are far from the corresponding significance thresholds.

4.2.3 Results for goal 3: minimise rank of a specific gene set

Random sample label permutations: Similar to the study on the Pickrell data set in the main document, we observe no over-optimistic results for GOSeq and clusterProfiler's ORA in the context of goal 3. In particular, the relative ranks in all permutations amount to 1 before and after the exploitation of uncertainty. This is consistent with the observation on the adjusted p-values of 1 observed for goal 2 (see Figure S3). For PADOG, we, again, observe moderate decreases in the ranks for the majority of permutations for both gene sets, all of which are triggered through the choice of pre-filtering or the method to transform the RNA-Seq data. As with the Pickrell data set, the ranks of both gene sets are moderately low for many of the permutations, therefore indicating a moderately high relevance to the condition of interest compared to the remaining gene sets. When inspecting the adjusted p-values, however, we observe that the level of adjusted p-values of both gene sets are generally high across all permutations. This indicates that the majority of the remaining gene sets in the GSA results are even higher (i.e. close to 1).

For clusterProfiler's GSEA, we observe that the relative ranks of both gene sets differ considerably between the different permutations before exploiting any uncertainties. In the majority of permutations, further decreases of moderate to notable magnitude can be obtained through various combinations of the analytical choices. However, for some of the permutations, we also observe that the relative rank and the corresponding adjusted p-value can differ considerably in magnitude. For instance, for gene set 2, we observe a decrease in the relative rank from 0.41 to 0.19 in one permutation. However, the corresponding adjusted p-values amount to 0.83 and 0.92, respectively. This

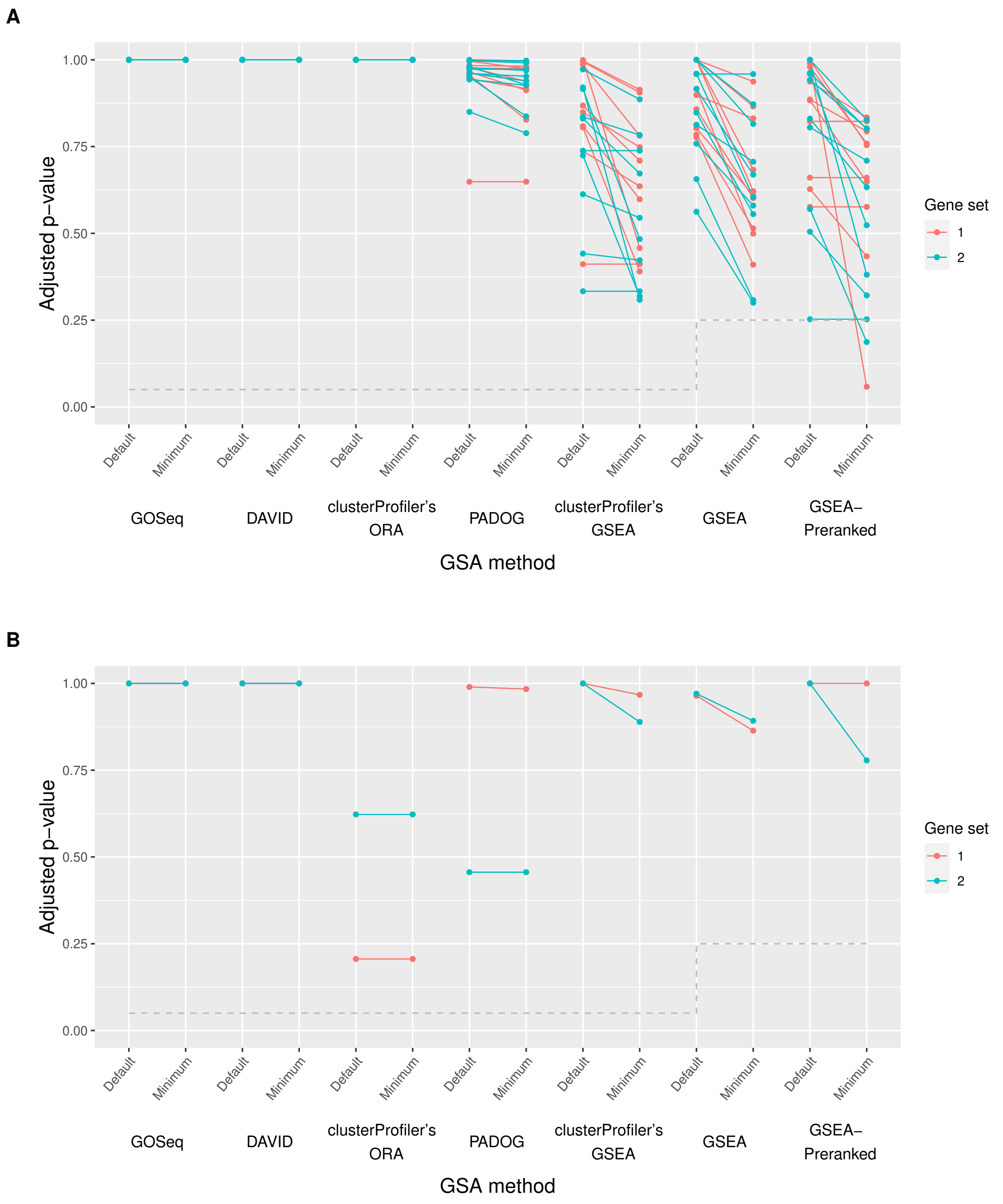

Figure S3: Goal 2: The optimised adjusted p-values (/q-values) in the Bottomly data set ('Minimum'), obtained through the exploitation of uncertainty, are compared to the corresponding values resulting from the default analytical choices ('Default'). Note that for the web-based applications `GSEA` and `GSEAPreranked`, the q-value is used to assess differential enrichment instead of the adjusted p-value. For each optimisation process, the associated optimised and the default adjusted p-value (/q-value) are connected through a line. (A) presents the results for the ten random permutations and (B) for true sample labels. On the x-axis, the individual methods investigated in the context of goal 2 are displayed. The results for gene set 1 are shown in red and those for gene set 2 in blue. The dashed grey line indicates the significance threshold for each method below which a gene set is considered differentially enriched.

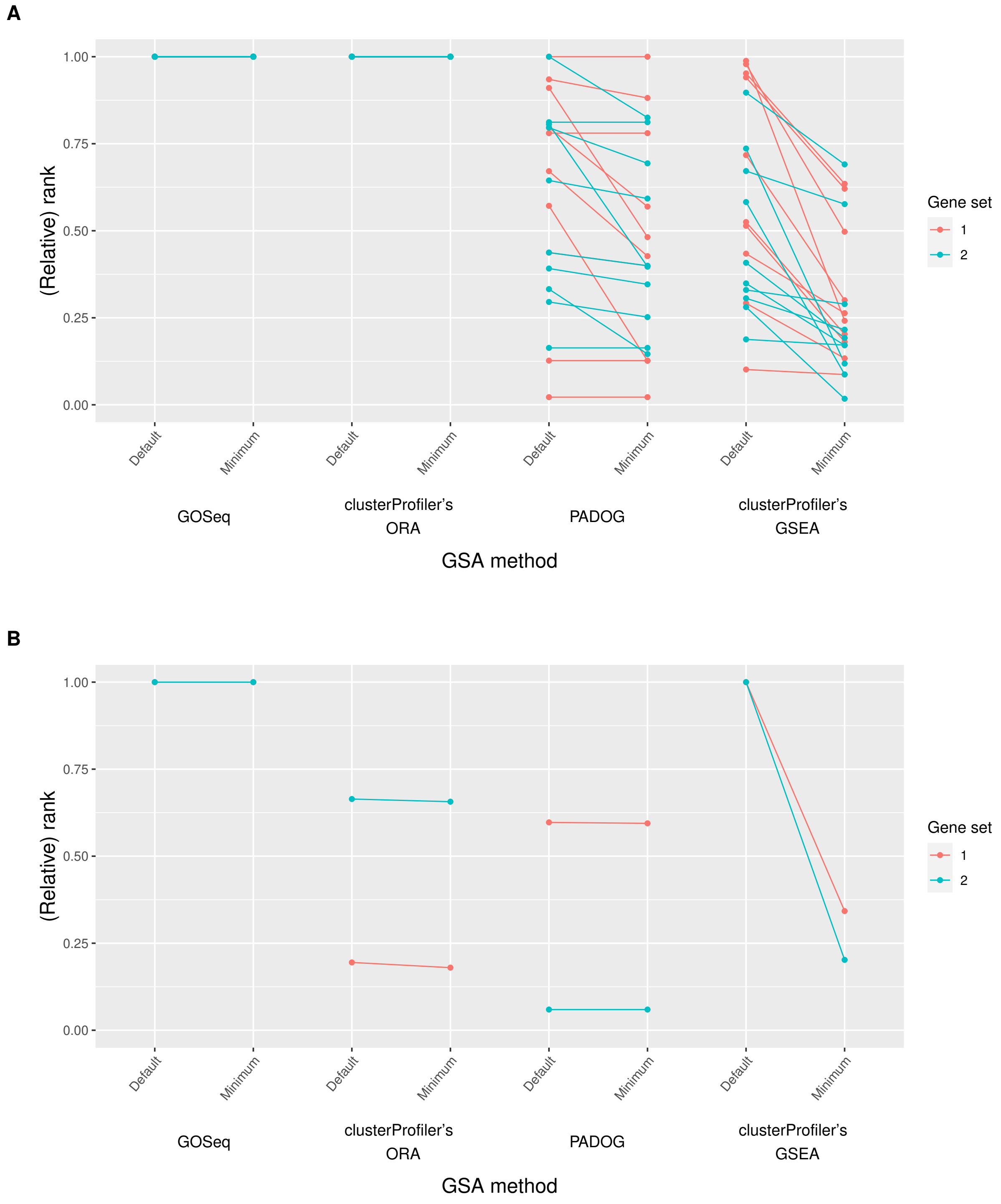

Figure S4: Goal 3: The (relative) ranks ('Minimum') in the Bottomly data set, obtained through the exploitation of uncertainty, are compared to the corresponding values resulting from the default analytical choices ('Default'). For each optimisation process, the associated optimised and the default rank are connected through a line. (A) presents the results for the ten random permutations and (B) for true sample labels. On the x-axis, the individual methods investigated in the context of goal 3 are displayed. The results for gene set 1 are shown in red and those for gene set 2 in blue.

indicates that the adjusted p-values of the remaining gene sets in the results table increase even more strongly through the exploitation of uncertainty than the adjusted p-value of gene set 2.

True sample labels:

We observe no to negligible decreases in the relative ranks of both gene sets for `GOSeq`, `clusterProfiler`'s ORA, and `PADOG`. For `clusterProfiler`'s GSEA, on the other hand, the ranks can be tweaked notably, namely from 1 to 0.34 and from 1 to 0.20 for gene sets 1 and 2, respectively. This implies a substantial increase in the 'relative' relevance of both gene sets to the condition of interest compared to the remaining genes in the corresponding results tables. However, the corresponding adjusted p-values remain high even after the exploitation of uncertainty. For instance, the tweaked rank of 0.2 of gene set 2 corresponds to an adjusted p-value of 0.98, indicating that the adjusted p-values of the remaining gene sets in the result table are even closer to 1. A similar observation is made for gene set 1.

References

Aleksander, S. A., Balhoff, J., Carbon, S., et al. (2023). The Gene Ontology knowledgebase in 2023. *Genetics*, 224(1):iyad031.

Ashburner, M. et al. (2000). Gene ontology: tool for the unification of biology. *Nature Genetics*, 25(1):25–29.

Bottomly, D., Walter, N. A., Hunter, J. E., Darakjian, P., Kawane, S., Buck, K. J., Searles, R. P., Mooney, M., McWeeney, S. K., and Hitzemann, R. (2011). Evaluating gene expression in C57BL/6J and DBA/2J mouse striatum using RNA-Seq and microarrays. *PloS one*, 6(3):e17820.

Hoffmann, S., Schönbrodt, F., Elsas, R., Wilson, R., Strasser, U., and Boulesteix, A.-L. (2021). The multiplicity of analysis strategies jeopardizes replicability: lessons learned across disciplines. *Royal Society Open Science*, 8(4):201925.

Kanehisa, M., Furumichi, M., Sato, Y., et al. (2023). KEGG for taxonomy-based analysis of pathways and genomes. *Nucleic Acids Research*, 51(D1):D587–D592.

Kanehisa, M. and Goto, S. (2000). KEGG: Kyoto Encyclopedia of Genes and Genomes. *Nucleic Acids Research*, 28(1):27–30.

Kraus, P., Xing, X., Lim, S. L., Fun, M. E., Sivakamasundari, V., Yap, S. P., Lee, H., Karuturi, R., and Lufkin, T. (2012). Mouse strain specific gene expression differences for Illumina microarray expression profiling in embryos. *BMC research notes*, 5(1):1–17.

Law, C. W., Chen, Y., Shi, W., et al. (2014). voom: precision weights unlock linear model analysis tools for RNA-Seq read counts. *Genome Biology*, 15(2):1–17.

Lopes-Ramos, C. M., Chen, C.-Y., Kuijjer, M. L., Paulson, J. N., Sonawane, A. R., Fagny, M., Platig, J., Glass, K., Quackenbush, J., and DeMeo, D. L. (2020). Sex differences in gene expression and regulatory networks across 29 human tissues. *Cell Reports*, 31(12).

Love, M. I., Huber, W., and Anders, S. (2014). Moderated estimation of fold change and dispersion for RNA-Seq data with DESeq2. *Genome Biology*, 15:550.

Robinson, M. D., McCarthy, D. J., and Smyth, G. K. (2010). edgeR: a Bioconductor package for differential expression analysis of digital gene expression data. *Bioinformatics*, 26(1):139–140.

Subramanian, A., Tamayo, P., Mootha, V. K., et al. (2005). Gene Set Enrichment Analysis: A knowledge-based approach for interpreting genome-wide expression profiles. *Proceedings of the National Academy of Sciences*, 102(43):15545–15550.

Wallenius, K. T. (1963). Biased sampling: the noncentral hypergeometric probability distribution. Technical report, PhD: Stanford University.

Wünsch, M., Sauer, C., Callahan, P., Hinske, L. C., and Boulesteix, A.-L. (2023). From RNA-Sequencing measurements to the final results: a practical guide to navigating the choices and uncertainties of gene set analysis. *arXiv preprint arXiv:2308.15171*.
